# Computer-Aided Drug Design and Synthesis of Rhenium Clotrimazole Antimicrobial Agents

**DOI:** 10.3390/antibiotics12030619

**Published:** 2023-03-20

**Authors:** Youri Cortat, Miroslava Nedyalkova, Kevin Schindler, Parth Kadakia, Gozde Demirci, Sara Nasiri Sovari, Aurelien Crochet, Stefan Salentinig, Marco Lattuada, Olimpia Mamula Steiner, Fabio Zobi

**Affiliations:** 1 Department of Chemistry, Fribourg University, Chemin Du Musée 9, 1700 Fribourg, Switzerland; 2 Haute école d’Ingénierie et d’Architecture, University of Applied Sciences Western Switzerland HES-SO, Pérolles 80, 1700 Fribourg, Switzerland; olimpia.mamulasteiner@hefr.ch

**Keywords:** rhenium, clotrimazole, *Staphylococcus aureus* MurG, docking

## Abstract

In the context of the global health issue caused by the growing occurrence of antimicrobial resistance (AMR), the need for novel antimicrobial agents is becoming alarming. Inorganic and organometallic complexes represent a relatively untapped source of antibiotics. Here, we report a computer-aided drug design (CADD) based on a ‘scaffold-hopping’ approach for the synthesis and antibacterial evaluation of *fac*-Re(I) tricarbonyl complexes bearing clotrimazole (ctz) as a monodentate ligand. The prepared molecules were selected following a pre-screening in silico analysis according to modification of the 2,2′-bipyridine (bpy) ligand in the coordination sphere of the complexes. CADD pointed to chiral 4,5-pinene and 5,6-pinene bipyridine derivatives as the most promising candidates. The corresponding complexes were synthesized, tested toward methicillin-sensitive and -resistant *S. aureus* strains, and the obtained results evaluated with regard to their binding affinity with a homology model of the *S. aureus* MurG enzyme. Overall, the title species revealed very similar minimum inhibitory concentration (MIC) and minimum bactericidal concentration (MBC) values as those of the reference compound used as the scaffold in our approach. The obtained docking scores advocate the viability of ‘scaffold-hopping’ for de novo design, a potential strategy for more cost- and time-efficient discovery of new antibiotics.

## 1. Introduction

One of the most concerning medical challenges nowadays consists in addressing the constant advent of new antimicrobial resistances (AMRs). Indeed, if this global health issue is not rapidly answered, the human cost could represent approximately ten million deaths a year in a near future [1]. For this purpose, there is an increasing need for novel drugs that could afford better activity against resistant pathogens by different mechanisms of action with respect to decades-old antibiotics. The testing of inorganic and organometallic complexes has arisen as an efficient strategy in order to overcome AMR [2,3,4,5,6,7,8,9,10], including coordination of well-established antimicrobial agents to a metal center [11]. The resulting inorganic and organometallic molecules are usually synthesized in a very straightforward manner and do not feature elaborated design, while often displaying superior biological activity compared to the coupled therapeutic potency of the drug or organic ligand and the metallic fragment. Moreover, the structural changes caused upon complexation commonly lead to distinct modes of action. This so-called ‘metal–drug synergism’ (MDS) occurs in particular thanks to improved pharmacokinetics through mutual chemical protection between the coordinated organic compound and the metal core [12].

A remarkable example of MDS in antibiotic research was reported by Sadler and coworkers, who synthesized piano stool Ir(III) complexes bearing various biguanide derivatives as bidentate ligand [13]. The complexes showed significant activity against multi-resistant *Staphylococcus aureus* (MRSA), with an MIC of 0.125 μg/mL (0.17 μM) alongside a fungicidal potency toward *C. albicans* and *C. neoformans* (MIC of 0.25 μg/mL), whereas uncoordinated biguanides displayed no activity within the tested range. Furthermore, no significant cytotoxicity was measured toward mammalian cells. Similar piano stool compounds [14,15] as well as iridium complexes with different scaffolds [16,17,18,19] were investigated by other groups with comparably promising results. Apart from iridium, gold, as a further example of a third-row transition metal, is popular and effective in antimicrobial research [20,21,22,23,24,25,26,27,28,29,30].

Our group [31,32,33], and others [34,35,36,37,38], have focused their attention on rhenium-based antibiotic agents [39]. Wenzel et al. first evaluated the antimicrobial potential of rhenium carbonyl complexes, reporting the activity of hetero-tri-organometallic compounds with MIC values of 1.4 µM against Gram-positive bacteria including MRSA [40]. The same groups also disclosed a family of *fac*-[Re(CO)_3_]^+^ *N*-heterocyclic carbene complexes with MIC values against Gram-positive bacteria between 0.7–2 µg/mL [41]. More recently, Cooper et al. introduced a new class of rhenium(V) dioxo complexes showing potent activity (MIC ≤ 0.25 μg mL^−1^) both against *Staphylococcus aureus* (*S. aureus*) and *Candida albicans* (*C. albicans*) alongside low human cell toxicity and low haemolytic action against human red blood cells [42]. Studies on the mechanism of action of these complexes are limited and possible biological targets still unknown. The hetero-tri-organometallic complexes of Wenzel et al. act on the cytoplasmic membrane of the bacteria, where they disrupt essential cellular processes as respiration and cell wall biosynthesis [40], while light-activated species of Frei et al. may induce microbe death via reactive oxygen species production and destabilization of Fe–S clusters [36].

**Figure 1:**
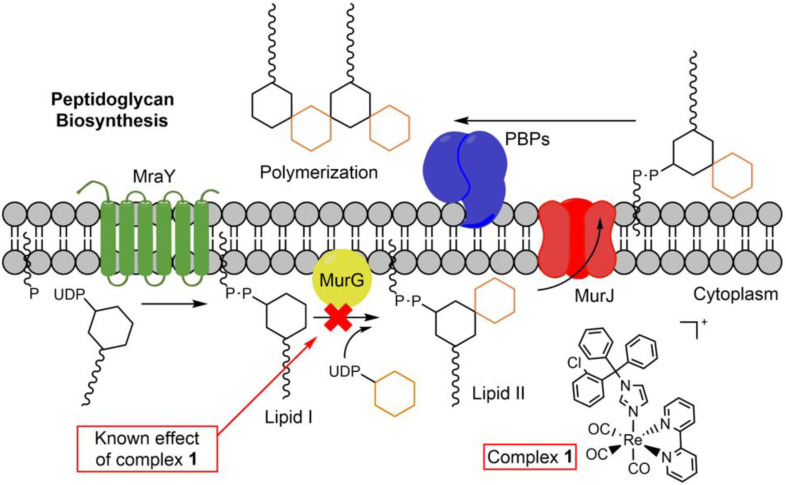
Schematic diagram of the membrane steps of the bacterial peptidoglycan synthesis pathway showing the known inhibitory function of **1**. For more details about the scheme, see [46].

Recently, however, Mendes et al. have studied in details the mechanism of action of a clotrimazole (ctz) complex of rhenium (complex **1, Figure 1**), showing that this compound acts as an inhibitor of membrane-associated stages of peptidoglycan synthesis [43]. Specifically, complex **1** triggers the accumulation of the ultimate cytoplasmic peptidoglycan precursor undecaprenyl-pyrophosphate-MurNAc-PP (Lipid I, Figure 1). The accumulation of Lipid I suggests an obstruction at a later stage of peptidoglycan biosynthesis. Mendes et al. showed that **1** inhibits undecaprenyl-pyrophosphate-MurNAc-PP-N-acetyl glucosamine biosynthesis (Lipid II, Figure 1) by inhibiting the MurG-mediated conversion of Lipid I to Lipid II. No effect on the MraY-mediated formation of Lipid I was observed. The authors further argued that inhibition of the MurG reaction can occur either by direct interference of the rhenium complex with the catalytic function of the enzyme or by sequestration of Lipid I serving as the substrate of MurG. It is unknown if **1** may sequester Lipid I, but there may be additional targets of **1** [43]. 

Overall, the study of Mendes et al. indicates that MurG may be selected as a target for the de novo drug design of antibiotic rhenium complexes. To this end, we have employed a computer-aided drug design (CADD) to identify new *fac*-[Re(CO)_3_]^+^ derivatives of ctz. CADD methods have played a key role in leading discovery in drug design research, adding complementing value to experimental high-throughput screening [44]. Nowadays, the use of virtual compound libraries affords access to unexploited regions of the chemical space [45], thereby extending the diversity of potential drug candidates, which will open new avenues in the search of new compounds labeled with sudden properties. Molecular de novo design enables a ‘scaffold-hopping’ approach in new drug discovery. In this contribution, we show our way of developing a combined experimental and computational methodological concept for finding new Re-ctz candidates with the desired antimicrobial effects. The concept of starting from known molecular skeletons as templates ensures significant chances for the new molecules with inspired scaffold variations to feature desirable antimicrobial properties.

## 2. Results and Discussion

### 2.1. De Novo Design—Pre-Screening Step

**Figure 2:**
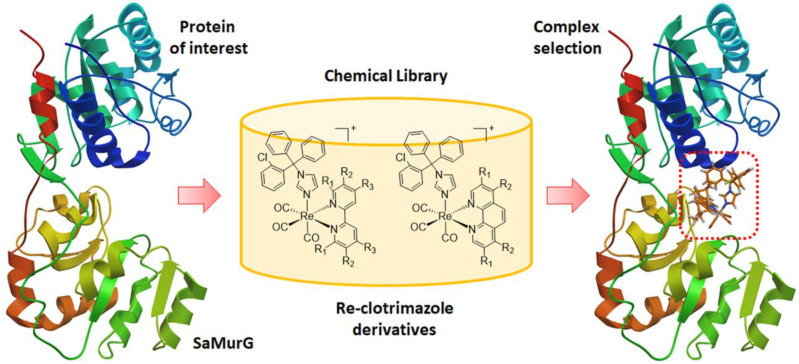
Schematic diagram of the conventional docking approach used to identify ‘hit’ complexes of Re-ctz derivatives.

**Figure 3:**
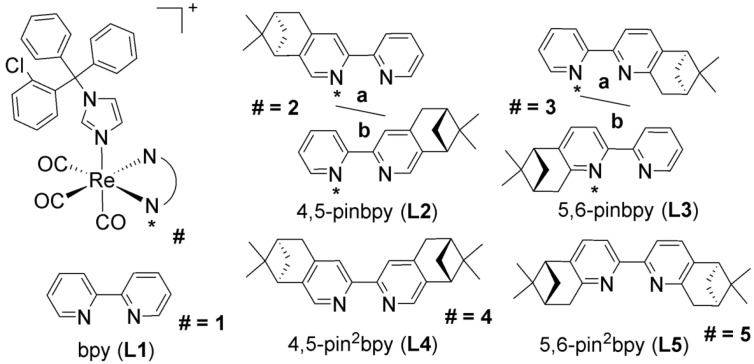
Molecular structure of Re-ctz complexes used for molecular docking with receptor SaMurG. Note that the # refers to the complexes. The corresponding 4,5-pinene and 5,6-pinene bipyridine are referred as L# in the text. The * refers to the specific nitrogen atom coordinated to the Re center as depicted for the complex on the top left.

In order to identify ‘hit’ derivatives of Re-ctz complexes, molecular design and initial scoring were performed in two steps. A pre-screening step was based on docking calculations without taking into account the flexibility of the receptor chains in the binding pocket, and then a refinement step following experimental results. The initial docking was performed using the homology models of *S. aureus* MurG and a chemical library of compounds (ca. 60 molecules) based on different diimine ligands previously selected to identify potent and non-toxic rhenium complexes active against *C. albicans*-MRSA co-infection [32] (Figure 2). Overall, the results indicated that, compared to the simple 2,2’-bipyridine (bpy) derivative of Mendes et al. (complex **1** in Figure 1), modification of the bidentate ligand did not improve the obtained docking scores of the new species, except where aliphatic substituents (i.e., a CH_3_ group) were introduced. In this case, the tested complexes improved by ca. 0.3–0.7 kcal mol^−1^ (depending on the homology model used). While not a significant improvement, rationalization of the data led us to eventually evaluate the possibility of testing 4,5-pinene and 5,6-pinene bipyridine derivatives (#,#-pinbpy in Figure 3) originally described by Hayoz and von Zelewsky [47]. When these ligands were introduced in the coordination sphere of the rhenium tricarbonyl species, and the corresponding ctz complexes were computationally evaluated, a significant increase (up to −1.7 kcal mol^−1^) in the docking scores of the complexes to homology models of *S. aureus* MurG (SaMurG) was calculated. 

Here we should mention that the results obtained from the docking scoring functions implemented in the Vina software do not represent a direct measure of high binding affinity. The calculated negative docking score is only considered here as indicative of a potentially better binding potency towards the selected receptor. The applied docking methods can predict multiple binding poses and calculate their docking score in a fast and computationally efficient way, and they are widely used to virtually screen potential drug candidates. However, the limitations of the applied methods should be addressed. The absolute values of the binding affinity obtained from docking programs should not be taken as the true values of binding affinity but rather could be used for obtaining possible lead structures and their binding poses. The effort in this field has focused in developing physical-based or knowledge-based docking scoring functions for efficiently predicting binding affinity with machine learning methods able to produce effective scoring functions [48,49,50]. However, it remains notoriously difficult to predict binding affinity by docking scoring functions. Warren et al. performed a study based on 10 docking programs and 37 scoring functions and the obtained results show that scoring functions need significant improvements for predicting binding affinity [51]. Other studies along the same line [52] compared the performance by ranking the binding of small molecules within seven scoring functions from five docking programs, one end-point method (MM/GBSA), and two MD-based free energy methods (PMX, FEP+). The obtained results are mostly in favor of the MD-based methods over the docking algorithms. 

The MD methods and the docking scoring functions are very different, because the scoring function assigns a common set of weights to the individual energy terms that contribute to the overall energy score. Many different types of scoring functions have been developed, although they often simplify the protein–ligand interactions on the complementarity shape and treat solvation effects implicitly, missing physical components such as entropy contributions from solvent molecules and proteins. Pose ranking is less accurate with docking methods and sometimes overlooks plausible binding poses. MD simulations provide the atomic resolution of protein structure and dynamics. With the free energy perturbation (FEP) or thermodynamic integration methods, all degrees of freedom relevant to protein–ligand bindings, including solvent motion and binding affinities, can be accurately predicted. The most stable binding poses of a given complex can be predicted accurately from long MD trajectories, in principle, even if high resolution structures of the complex are unavailable. The protein flexibility is not sufficiently considered in most of the docking simulations, while ligand bindings often couple with protein conformational changes. Docking methods are, therefore, less accurate in ranking the predicted poses and sometimes overlook plausible binding structures. In our case, the main limitation for applying MD simulation to Re complexes is that there are no bonding and non-bonding parameters available in the force field implemented in the commonly used MD codes. Thus, docking calculations appear to us as the only possible and reasonable choice to accomplish the objective set out in the present study.

### 2.2. Synthesis and Characterization of Complexes

**Scheme 1:**
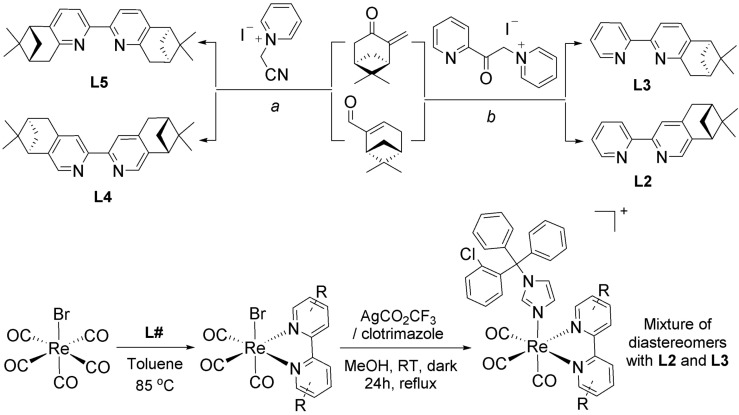
Top and bottom respectively: general synthetic procedures and chemical structures of 4,5-pinene and 5,6-pinene bipyridine ligands (L#) and of Re complexes. For ligand synthesis, conditions are: (*a*) stepwise 1. EtOH/AcOH NH_4_OAc; 2. NaNO_2_, HBr, 0°; 3. CuBr, HBr, 70°; 4. Zn/[Ni(PPh_3_)_4_Cl_2_], DMF [55]. (*b*) NH_4_OAc/AcOH, 100° [47].

Following prescreening identification of the new potential antibiotic complexes, we moved to the preparation of the candidate molecules. The 4,5-pinene and 5,6-pinene bipyridine ligands (**L2**–**L5** in Scheme 1) were synthesized according to established procedures [47,53]. Ligands **L2** and **L3** were prepared in a single step from the reaction of natural (lR)-(-)-myrtenal (**L2**) or 1R-(+)-pinocarvone (**L3**) [54] with 1-(2-oxo-2-(pyridin-2-yl)ethyl)pyridin-1-ium iodide. Ligands **L4** and **L5** were prepared from the same starting materials, following their condensation with 1-(cyanomethyl)pyridin-1-ium iodide according to the chemistry described by Fletcher et al. [55]. Finally, the rhenium complexes were obtained by treatment of *fac*-[Re(CO)_5_Br] with the corresponding ligand **L#** in hot toluene, followed by abstraction of the coordinated bromide and reaction with the drug ctz (Scheme 1).

IR spectroscopy analysis was in accordance with the typical tricarbonyl vibration pattern for this type of species. ^1^H NMR spectra of the complexes showed pure diamagnetic compounds, according to the symmetry given by the facial-arranged CO’s and low-spin d^6^ nature of the metal ion. Given the chiral nature of **L2** and **L3**, Re complexes of those ligands (i.e., compounds **2** and **3**) were obtained in a 3:1 (**2**) or 4:1 (**3**) mixture of diastereomers (as determined by ^1^H NMR analysis). Attempts to isolate the two different diastereomers were unsuccessful, thus in order to identify the relative ratio of species **2a**:**2b** and **3a**:**3b**, we performed DFT calculations. As perhaps intuitively expected, the computational data indicate that diastereomers **2a** and **3a** (i.e., diastereomers with the two pinene methyl groups pointing towards the opposite plane of the ctz ligand) are more stable than the corresponding species **2b** and **3b** by 0.6 and 0.8 kcal/mol, respectively. It must be said that the energy difference is minimal, thus it is difficult to state if indeed the samples are enriched in the **#a** diastereomers.

**Figure 4:**
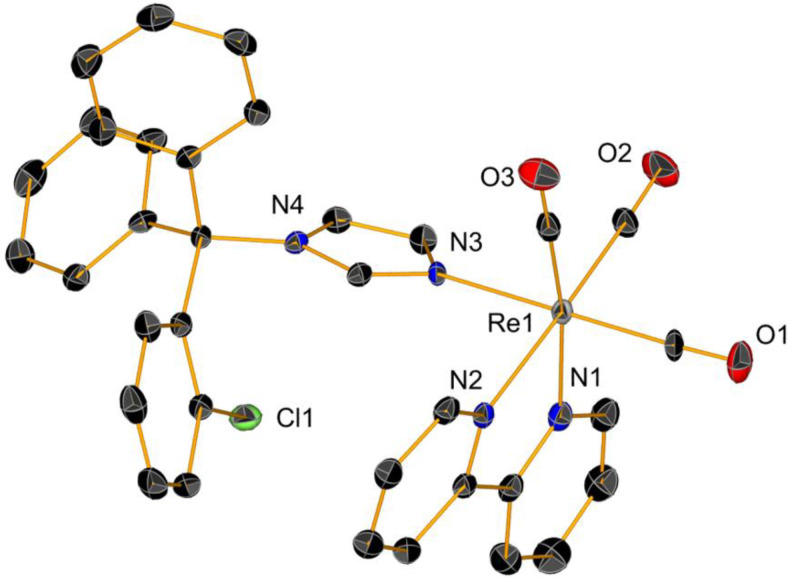
Crystal structures of complex **1**. Thermal ellipsoids are at 30% probability. Hydrogen atoms are omitted for clarity.

We were unsuccessful in obtaining crystals of complexes **2**–**5**, but crystals of complex **1** suitable for X-ray diffraction measurements were obtained by diffusion of diethyl ether into a solution of the compound in acetonitrile at −20 °C. The ctz complex **1** (Figure 4) represents only the third example of a metal carbonyl species bearing a coordinated azole that is structurally characterized. The structure of **1** is similar to the corresponding *fac*-[Mn(CO)_3_(bpy)]^+^ complex [11], but its crystallographic parameters are significantly dissimilar. In comparison to the Mn analogue, the X-ray structure analysis of **1** shows that metrical parameters around the central Re ion define a more regular octahedron, and that the five-membered ring of the ctz ligand is not tilted toward the bpy ring plane. The Re and Mn structures share a common feature, represented by a weak interaction between one of the ctz phenyl rings and one of the aromatic rings of the bpy ligand (Figure S17, Supplementary Material). A second structure, featuring the *fac*-[Re(CO)_3_(phen)]^+^ unit (where phen = 1,10-phenanthroline), was recently reported by Soba et al. in a study that investigated the potential of the complex as a bioactive agent for the treatment of Chagas disease [56]. Other structurally characterized organometallic complexes of ctz are relatively rare. Such compounds are known only for platinum [57], ruthenium [58,59], and rhodium [60]. The other reported structures featuring a ctz moiety are of purely inorganic nature with copper, zinc [61,62,63], palladium [64], and gold [60] metal centers. 

### 2.3. Antimicrobial Activity and Refined Docking Studies of Complexes

**Figure 5:**
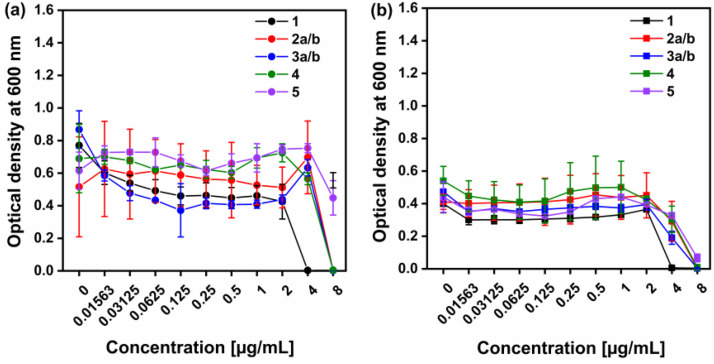
Antibacterial activity of different Re-ctz complexes against (**a**) *S. aureus* wild-type strain, and (**b**) *S. aureus* methicillin-resistant type strain, respectively.

Following the initial screening results and synthesis of complexes, in the next step, we moved to the antimicrobial evaluation of the molecules. The results of this study positively advocate that the approach for the ‘scaffold-hopping’ design leads to very good results, which were confirmed with the MIC values presented in Table 1. The antibacterial properties of the Re-based compounds were investigated in vitro by measuring the MIC (Figure 5) and the minimum bactericidal concentration (MBC, Table 1) against two *S. aureus* strains (wild type and methicillin-resistant), respectively. Following the Clinical and Laboratory Standards Institute (CLSI) guidelines, the MIC is the lowest concentration of an antimicrobial agent inhibiting the visible growth of the bacteria after 24 h treatment [65]. The MBC represents the bactericidal endpoint of an antimicrobial agent and is defined as >99.9% (>3-log) reduction of the initial inoculum after 24 h treatment [65]. Complexes **2a**/**b**, **3a**/**b**, and **4** had an MIC of 8 µg mL^−1^ against both *S. aureus* strains while complex **5** had an MIC > 8 µg mL^−1^ (Table 1). 

In comparison, complex **1** had an MIC of 4 µg mL^−1^. DMSO used as a negative control did not inhibit the growth of the bacteria and had an MIC > 8 µg mL^−1^. The results showed that the newly synthesized Re-ctz complexes were active against the tested bacterial strains, and with very similar MIC and MBC values to the parent complex **1**. Here it should be pointed out that a random diimine ligand variation of equivalent Mn complexes invariably lead to inactive molecules [43]. Our results thus confirm that our CADD grounded on a ‘scaffold-hopping’ approach is a valuable methodology for a de novo drug design based on a molecular scaffold from compounds with already proven antimicrobial properties.

**Figure 6:**
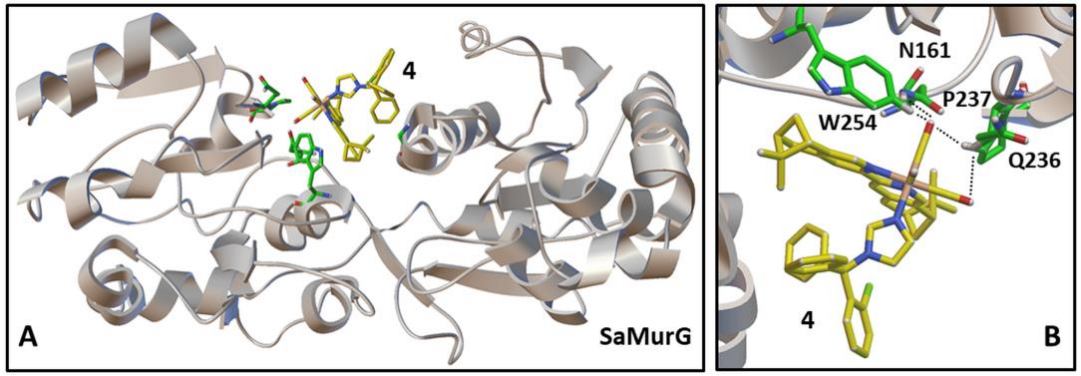
Full (**A**) and detail (**B**) view of the binding region of computer-generated lowest energy pose of complex **4** in SaMurG homology model.

The antimicrobial data were finally compared with the refined molecular docking of the tested ligands. We found that compound **4** exhibited the best binding towards the SaMurG receptor, while the compounds **2a** and **5** the lowest docking score within the screened set. The ligand–receptor coordination of compound **4** may be explained by the strong interaction with the binding site through the formation of H-bonds (HBs) with important amino acids. The binding energy for **4** was found to be −8.86 kcal/mol, and it interacted with the binding pocket of SaMurG by forming seven HBs with GLN 15 and 236, PRO 237, TRP 254, GLY 15, and ANS 161. Figure 6 shows the interactions between the compound **4** and selected amino acid residues in the binding pocket. The detailed amino acids environment distribution of the active site of the receptor are presented in the Supporting Information for each of the compounds and interactions listed in Table 2. Compounds **3a** and **3b** showed lower docking scores (−7.6 and −7.4 kcal/mol) and formed less HBs with the receptor. The observed behavior could be related to the change in the amino acid’s environment interaction modes of the ligands with the receptor, and particularly with the hydrophilic amino acid backbone in the binding site.

**Figure 7:**
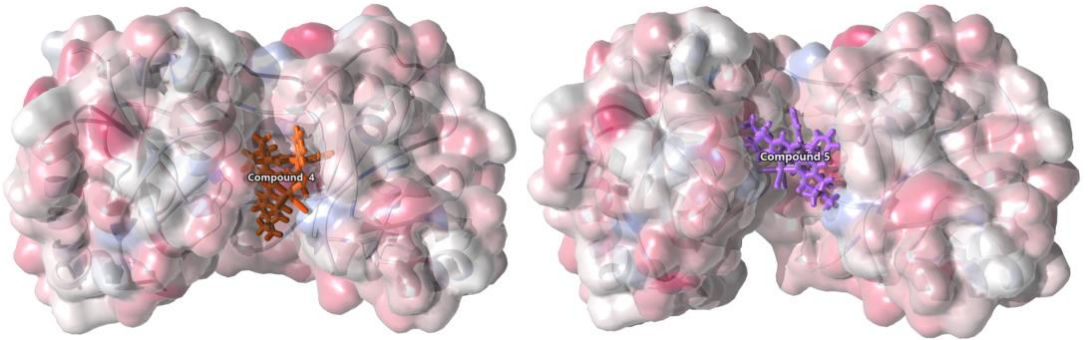
Representation of the structure of the homological model for SaMurG based on the residue hydrophobicity.

The molecular docking of the new designed structures was used to study binding interactions in the active site of the homological model of SaMurG. Based on the results, we have a compound with a high docking score, with the nonpolar contributions from the amino acids environment stabilizing the binding affinity of the ligand **4** in the binding pocket. Figure 7 shows a comparison of the lowest energy-binding pose of **4** and **5** with SaMurG displaying hydrophobicity scales of the amino acids in the binding cavity of the pocket. It is undeniable that the distributed networks of the ligand–receptor HB play a significant role in the process of receptor inhibition. As a result of the findings that were presented in the study, it is expected that further research and extended libraries with new compounds will be conducted towards developing a new drug specifically designed to treat antimicrobial resistance problems. 

## 3. Conclusions

Initial CADD calculations based on a ‘scaffold-hopping’ approach allowed designing complexes **2**–**5** as potentially active agents against *S. aureus* by comparison of their docking scores toward SaMurG with the already known bpy derivative **1**. Molecules were successfully synthesized and suitable crystals for X-ray diffraction of **1** were successfully grown. The pre-synthetic results were corroborated by subsequent biological assays in which most compounds displayed MIC and MBC values in the low micromolar range comparable to **1**, even toward the resistant *S. aureus* strain. In our case, the functionalization of the bpy ligand at positions 4 and 4′ did not seem to significantly impact the antibacterial activity of the resulting complex like it was proven to occur for manganese analogs [43]. Indeed, further in silico investigation using a refined algorithm for molecular docking confirmed the high affinity of all title species for SaMurG, especially in the case of compound **4**, which features stabilizing nonpolar interactions in the binding pocket. Undoubtedly, one of the main roles was played by the distributed networks of the ligand–receptor HBs. In summary, the outcomes of the present study afforded evidence toward the viability of the de novo drug design strategy. Indeed, this method allowed us to identify relevant drug candidates, validate their antimicrobial ability, and confirm affinity properties required in order to undergo further design and studies aimed at their mechanism of action.

## 4. Materials and Methods

### 4.1. Reagents, Instruments, and Analysis

Solvents and reagents were obtained from standard suppliers and used as received. The compound [Re(CO)_5_Br] was purchased from Sigma-Aldrich, while the drug ctz from Thermoscientific-Acros. Compound **1** [43] and ligands (6R,8R)-7,7-dimethyl-3-(pyridin-2-yl)-5,6,7,8-tetrahydro-6,8-methanoisoquinoline (4,5-pinbpy), (5R,7R)-6,6-dimethyl-2-(pyridin-2-yl)-5,6,7,8-tetrahydro-5,7-methanoquinoline (5,6-pinbpy) [47], 4,5-pin^2^bpy, and 5,6-pin^2^bpy [53,55] were prepared according to published procedures. Unless otherwise noted, the solvents used in the preparation of all molecules were dry and O_2_-free. NMR spectra were measure on a Bruker Advance III 400 MHz spectrometer. ^1^H chemical shifts of molecules are reported relative to the residual solvent protons. Mass analysis measurements were carried out with a Bruker FTMS 4.7-T Apex II spectrometer; UV-vis spectra on a Thermo Fisher Scientific Jasco V730 spectrophotometer; InfraRed spectra (solid state) on a Bruker TENSOR II spectrometer (4000–600 cm^−1^ range, 4 cm^−1^ resolution). Single crystals suitable for X-ray diffraction were measured with a Stoe IPDS2 diffractometer (CuKα1 radiation) furnished with an Oxford Cryosystems mechanical cooler. Structure solution: The ShelXT Crystal program was used to solve and refine the structures [66,67]. Crystallographic data, CCDC number 2244172, have been deposited at the Cambridge Crystallographic Data Centre.

### 4.2. Synthetic Procedures

Complexes **1–5** [32] were synthesized by reacting [Re(CO)_5_Br] (1.0 equiv.), with the desired bipyridine (bpy) ligand (1.0 equiv.) in hot toluene (refluxing temperature) for 7–9 h. Upon cooling of the solution (RT), the corresponding *fac*-[Re(CO)_3_(#,#-pinbpy)Br] (obtained as a fluorescent yellow powder) was filtered and washed twice with cold toluene as. The solid was then desiccated under vacuum for 24 h. NMR and HPLC measurements indicated a purity of ≥96%. They were then suspended in MeOH (0.02 M) with ctz (1.5 equiv.) and AgOTf (2.0 equiv.) and refluxed in the dark for 20 h. After the reaction mixture had cooled down to the room temperature, it was filtered to discard AgBr and dried in vacuo. The crude products were purified by semi-preparative HPLC.

*fac*-[Re(CO)_3_(4,5-pinbpy)ctz] (**2**). Yellow solid, yield 13%. IR (solid, cm^−1^); ν_CO_: 2027, 1902. UV-Vis (MeOH), λ_max_ [nm]: 327, 315. ^1^H-NMR (400 MHz, CD_3_CN, ppm): 9.00 (d, *J* = 5.50 Hz, 1H; **a**) 8.96 (d, *J* = 5.50 Hz, 1H; **b**) 8.59 (s, 0.1H; **a**) 8.52 (s, 1H; **b**) 8.24 (d, *J* = 8.07 Hz, 1H; **a**/**b**) 8.14–8.19 (m, 1H; **a**/**b**) 8.10 (s, 1H; **a**/**b**) 7.52–7.59 (m, 1H; **a**/**b**) 7.23–7.45 (m, 10H; **a**/**b**) 6.99 (t, *J* = 1.28 Hz, 1H; **a**/**b**) 6.79–6.83 (m, 6H; **a**/**b**) 6.70 (s, 1H; **a**) 6.62 (s, 1H; **b**) 3.15–3.22 (m, 2H; **a**/**b**) 2.96–3.03 (m, 1H; **a**/**b**) 2.72–2.82 (m, 1H; **a**/**b**) 2.35–2.39 (m, 1H; **a**/**b**) 1.44 (s, 3H; **a**) 1.42 (s, 3H; **b**) 1.21 (d, *J* = 10.03 Hz, 1H; **b**) 1.00 (d, *J* = 10.03 Hz, 1H; **a**) 0.66 (s, 3H; **a**) 0.42 (s, 3H; **b**). ESI^+^-MS (MeOH): *m*/*z*, 864.9 [C_42_H_35_ClN_4_O_3_Re]^+^, [M]^+^.

*fac*-[Re(CO)_3_(5,6-pinbpy)ctz] (**3**). Pale yellow solid, yield 24%. IR (solid, cm^−1^); ν_CO_: 2027, 1906. UV-Vis (MeOH), λ_max_ [nm]: 334. ^1^H-NMR (400 MHz, CD_3_CN, ppm): 9.06 (d, *J* = 5.50 Hz, 1H; **a**) 9.01 (d, *J* = 5.50 Hz, 1H; **b**) 8.03–8.27 (m, 3H; **a**/**b**) 7.70–7.73 (m, 1H; **a**/**b**) 7.53–7.62 (m, 1H; **a**/**b**) 7.23–7.45 (m, 10H; **a**/**b**) 7.05 (s, 1H; **b**) 6.94 (s, 1H; **a**) 6.77–6.86 (m, 7H; **a**/**b**) 6.60 (s, 1H; **a**) 3.45 (d, *J* = 2.81 Hz, 1H; **a**/**b**) 2.99 (t, *J* = 5.75 Hz, 1H; **a**/**b**) 2.70–2.78 (m, 1H; **a**/**b**) 2.47–2.51 (m, 1H; **a**/**b**) 1.43 (s, 3H; **a**) 1.41 (s, 3H; **b**) 1.28–1.30 (m, 1H; **a**/**b**) 0.95 (d, *J* = 10.15 Hz, 1H; **a**) 0.64 (s, 3H; **a**) 0.38 (s, 3H; **b**). ESI^+^-MS (MeOH): *m*/*z*, 864.9 [C_42_H_35_ClN_4_O_3_Re]^+^, [M]^+^.

*fac*-[Re(CO)_3_(4,5-pin^2^bpy)ctz] (**4**). Yellow solid, yield 8.0%. IR (solid, cm^−1^); ν_CO_: 2025, 1898. UV-Vis (MeOH), λ_max_ [nm]: 332, 282. ^1^H-NMR (400 MHz, CD_3_CN, ppm): 8.54 (s, 1H) 8.46 (s, 1H) 8.06 (d, *J* = 7.70 Hz, 2H) 7.46 (td, *J* = 7.95, 1.47 Hz, 1H) 7.24–7.40 (m, 9H) 7.08 (t, *J* = 1.47 Hz, 1H) 6.87 (t, *J* = 1.59 Hz, 1H) 6.76–6.83 (m, 5H) 6.57 (s, 1H) 3.09–3.21 (m, 4H) 2.99 (t, *J* = 5.38 Hz, 1H) 2.94 (t, *J* = 5.26 Hz, 1H) 2.70–2.81 (m, 2H) 2.34–2.38 (m, 2H) 1.41–1.46 (m, 7H) 1.22 (d, *J* = 10.03 Hz, 1H) 0.97 (d, *J* = 10.03 Hz, 1H) 0.66–0.70 (m, 4H) 0.41 (s, 3 H). ESI^+^-MS (MeOH): *m*/*z*, 959.0 [C_49_H_45_ClN_4_O_3_Re]^+^, [M]^+^.

*fac*-[Re(CO)_3_(5,6-pin^2^bpy)ctz] (**5**). Pale yellow solid, yield 15%. IR (solid, cm^−1^); ν_CO_: 2025, 1900. UV-Vis (MeOH), λ_max_ [nm]: 343, 286. ^1^H-NMR (400 MHz, CD_3_CN, ppm): 7.97 (t, *J* = 7.58 Hz, 2H) 7.67 (t, *J* = 8.74 Hz, 2H) 7.24–7.44 (m, 10H) 6.97 (t, *J* = 1.53 Hz, 1H) 6.79–6.88 (m, 6H) 6.53 (t, *J* = 1.34 Hz, 1H) 3.42–3.60 (m, 4H) 2.95–2.99 (m, 2H) 2.70–2.76 (m, 2H) 2.48–2.52 (m, 2H) 1.43 (s, 3H) 1.42 (s, 3H) 1.26 (d, *J* = 10.03 Hz, 1H) 0.97 (d, *J* = 10.03 Hz, 1H) 0.63 (s, 3H) 0.45 (s, 3H). ESI^+^-MS (MeOH): *m*/*z*, 959.1 [C_49_H_45_ClN_4_O_3_Re]^+^, [M]^+^.

### 4.3. Antimicrobial Assay

The antimicrobial activity of different Re-ctz complexes was assessed against *Staphylococcus aureus* ATCC 35556 [wild type] and *Staphylococcus aureus* ATCC 43300 (methicillin-resistant) strains following the Clinical and Laboratory Standards Institute (CLSI) guidelines [65] and the broth microdilution protocol of Wiegand et al. [68]. Briefly, 16 µg mL^−1^ of each complex was prepared in DMSO as a stock solution and was UV-sterilized for 30 min before use. Each stock solution was twofold diluted in PBS and 50 µL of each dilution was dispatched in 96-well plates. In parallel, *S. aureus* overnight cultures grown in Mueller–Hinton Broth (non-cation-adjusted, MHB) were used to prepare bacterial suspensions at 1 × 10^6^ CFU mL^−1^ in MHB 2×. Next, 50 µL of *S. aureus* suspensions was mixed in the 50 µL of complexes serial dilutions, leading to a final bacterial concentration of 5 × 10^5^ and the complexes concentrations ranging from 8 µg ml^−1^ to 0.01563 ug mL^−1^. The microtiter plates were then incubated at 37 °C for 24 h. The minimum inhibitory concentration (MIC) values were determined by measuring the optical density at 600 nm (OD_600_). The minimum bactericidal concentration (MBC) values were determined by spotting 5 µL of each dilution on MHB agar plates, and CFU counting after overnight incubation at 37 °C. The assay was conducted in triplicate.

### 4.4. In Silico Calculations

#### 4.4.1. Structural Modeling of *S. aureus* MurG—Initial Homological Model for Pre-Screened Study

The crystal structure of the *S. aureus* MurG protein is not reported; thus, to perform docking calculations, we employed different homology models. The initial screening of compounds was performed with *E. coli* MurG [69] and homology models built according to Mann et al. [70], the protein homology/analogy recognition engine (Phyre^2^) [71], the UniProt sequence Q6GGZ0 [72]. The Phyre^2^ engine identified the crystal structure of the *P. aeruginosa* MurG as the most suitable template for the 3D homology model [73]. In agreement with what was previously reported [70], our homology model predicts a high structural relation between *S. aureus* MurG (SaMurG) and *E. coli* MurG (EcMurG) [69]. Cross analysis of the folding results was performed with the (PS)^2^ protein structure prediction server (version 3.0) [74]. The server also identified the *P. aeruginosa* MurG as the most suitable template, but provided a slightly different predicted 3D structure of SaMurG with a β-sheet close to the proposed membrane binding interface [75]. The docking calculations were performed as previously described [31] with the exception that the receptors were kept rigid. Preliminary molecular docking calculations were carried out with the AutoDock Vina (version 1.2.0) [76] and the AutoDock4 (version 4.2.6) [77] softwares. The Biovia Discovery Studio Visualizer software (version 21.1.0.20298) was employed to distinguish interactions between the ligand and the receptor.

#### 4.4.2. Homological Modeling with ESMFold from Meta and Molecular Docking Simulations

The homology modeling for building the unknown protein 3D structure for *S. aureus* MurG protein was primary checked by the sequence scoring function based on similarity exploring with a Swiss model based on *E. coli* MurG. Regarding results for the structural scoring similarities with other proteins, we defined the following with highest similarity score PDB: 1NLM. Then, the overlapping score was recalculated with ESMFold implemented in the Samson (https://www.samson-connect.net, accessed on 21 November 2021, Figure 8). The process of homology modeling was conducted in sequential steps. The best model was obtained with Samson, then the docking was conducted. The hydrogens close to ligands were not fixed, nor were the whole set of chains in the binding pocket. The next step was docking the ligand molecules with Autodock Vina implemented in Samson elements [78,79].

#### 4.4.3. DFT Calculations

All computations were performed with the Gaussian 09 programs. Geometry optimizations as well as frequency analysis calculations were performed in the gas phase. The Becke three-parameter hybrid functional B3LYP [80] for H, C, O, and N was used in combination with the standard SDD basis sets for Re [81,82,83] and Perdew–Burke–Ernzerhof (PBE) [84,85] auxiliary basis set with Planewave. Geometries optimizations were performed without symmetry restrictions. Vibrational frequencies were computed to verify minima. We observed no imaginary frequencies for the reported structures and values.

## Figures and Tables

**Figure 8:**
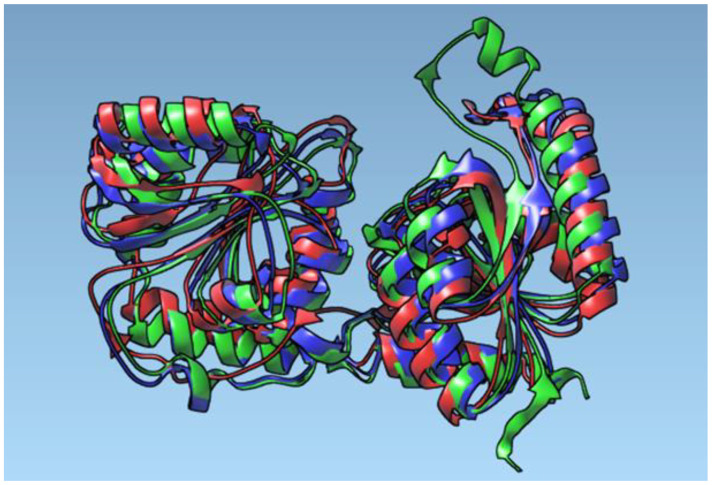
Comparison between the PBD: 1NLM and new model. Aligned models using green—predicted structure with ESMFold (implemented in SAMSON); red—Swiss model server and blue—PDB: 1NLM.

**Table 1:** MIC and MBC values of Re-ctz complexes against *S. aureus* strains.

Complexes	MIC [µg mL^−1^]		MBC [µg mL^−1^]	
	Wild-Type	Resistant-Type	Wild-Type	Resistant-Type
**1**	4	4	8	8
**2a**/**b**	8	8	>8	>8
**3a**/**b**	8	8	>8	>8
**4**	8	8	8	8
**5**	>8	>8	8	8
DMSO (negative control)	>8	>8	>8	>8
Vancomycin **[31]**	9	9	-	-

**Table 2:** Molecular docking results for the homological model MurG inhibitors **1**–**5**.

Compound	Docking Score (kcal/mol)	Receptor—Ligand Contact Area [nm^2^]	H Bonds	Receptor AA	Ligand Atom	Donor—Acceptor Dostance [Å]	H-A Distance [Å]	D-H Bond Length [Å]	Angle
1 *	−8.06	4.3	2	ARG 67 ARG 67	Re-CO Re-CO	3.37 3.26	2.49 2.33	1.00 1.00	146.35 154.04
**2a**	−7.20	5.1	0						
**2b**	−8.0	4.9	3	GLY 14 GLY 15 GLU 47	pin-CH ctz-Ph ctz-Ph	3.48 3.49 3.30	2.66 2.63 2.56	1.08 1.11 1.08	131.89 134.01 124.72
**3a**	−7.6	5.1	3	ARG 67 ARG 67 GLN 127	Re-CO Re-CO bpy-CH	3.19 3.18 3.40	2.30 2.27 2.40	1.00 1.00 1.09	148.53 150.33 151.84
**3b**	−7.4	5.1	2	GLY 18 GLY 18	ctz-Ph ctz-Ph	3.49 3.27	2.64 2.41	1.08 1.08	135.46 135.68
**4**	−8.86	5.2	7	GLY 15 ANS 161 GLN 236 GLN 236 GLN 236 PRO 237 TRP 254	pin-CH Re-CO Re-CO Re-CO Re-CO Re-CO Re-CO	3.35 3.2 3.41 3.37 3.50 3.43 3.42	2.31 2.41 2.67 2.65 2.78 2.39 2.65	1.11 1.00 1.09 1.08 1.08 1.09 1.08	156.17 140.60 124.42 123.92 123.29 159.37 127.79
**5**	−7.25	5.3	1	MET 103	Re-CO	3.50	2.61	1.09	138.85

* Reference compound.

## Data Availability

Data available upon request.

## Supplementary Materials

The following supporting information can be downloaded at: https://www.mdpi.com/article/10.3390/antibiotics12030619/s1, Figure S1: ^1^H NMR spectrum of a **2a/2b** 3:1 mixture in CD_3_CN, ✱ = solvent residual peak; Figure S2: ^1^H NMR spectrum of a **2a/2b** 3:1 mixture in CD_3_CN, aromatic region; Figure S3: ^1^H NMR spectrum of a **2a/2b** 3:1 mixture in CD_3_CN, aliphatic region; Figure S4: ^1^H NMR spectrum of a **3a/3b** 4:1 mixture in CD_3_CN, ✱ = solvent residual peak; Figure S5: ^1^H NMR spectrum of a **3a/3b** 4:1 mixture in CD_3_CN, aromatic region; Figure S6: ^1^H NMR spectrum of a **3a/3b** 4:1 mixture in CD_3_CN, aliphatic region; Figure S7: ^1^H NMR spectrum of **4** in CD_3_CN; Figure S8: ^1^H NMR spectrum of **4** in CD_3_CN, aromatic region; Figure S9: ^1^H NMR spectrum of **4** in CD_3_CN, aliphatic region; Figure S10: ^1^H NMR spectrum of **5** in CD_3_CN; Figure S11: ^1^H NMR spectrum of **5** in CD_3_CN, aromatic region; Figure S12: ^1^H NMR spectrum of **5** in CD_3_CN, aliphatic region; Figure S13. IR spectrum of a **2a**/**2b** 3:1 mixture (left) and of a **3a**/**3b** 4:1 mixture; Figure S14. IR spectrum of **4** (left) and **5**; Figure S15. UV-Vis spectrum of a **2a**/**2b** 3:1 mixture (left) and of a **3a**/**3b** 4:1 mixture in MeOH; Figure S16. UV-Vis spectrum of **4** (left) and of **5** in MeOH; Figure S17. Weak interaction between one of the ctz phenyl rings and one of the aromatic rings of the bpy ligand in the structures of **1** (left) and corresponding Mn complex; Figure S18. Homological model Residues in the pocket; Figure S19. Surrounding amino acids histogram for Drug **1** and 2D distribution bonds in two planes (YZ and XY) for the H-bonds from the center of the ligand; Figure S21. Surrounding amino acids histogram for Drug **2a** and H-bonds distribution maps in two planes (ZY and XY); Figure S22. Surrounding amino acids histogram for Drug **2b** and H-bonds distribution map in plane YZ; Figure S23. Surrounding amino acids histogram for Drug **3a** and H-bonds distribution map map for plane YZ; Figure S24. Surrounding amino acids histogram for Drug **3b** and H-bonds distribution map in plane YZ; Figure S25. Surrounding amino acids histogram for Drug **3b** and H-bonds distribution map for plane YZ; Table S1. Prescreening AutoDock molecular docking results for different homological MurG models (fixed receptor) with inhibitors **1**–**5**.
