# Supplementary material for: Computer-Aided Drug Design and Synthesis of Rhenium Clotrimazole Antimicrobial Agents
Source: Antibiotics (Basel). 2023 Mar 20;12(3):619. doi: 10.3390/antibiotics12030619 (PMC10044843; doi:10.3390/antibiotics12030619)
Supplement: Supplementary file 1 [file antibiotics-12-00619-s001.zip › antibiotics-2280159-supplementary.pdf]

Supplementary Information for

Computer-aided drug design and synthesis of rhenium clotrimazole antimicrobial agents

Youri Cortat [1], Miroslava Nedyalkova [1], Kevin Schindler [1], Parth Kadakia [1], Gozde Demirci [1], Sara Nasiri Sovari [1], Aurelien Crochet [1], Stefan Salentinig [1], Marco Lattuada [1], Olimpia Mamula Steiner [2,*] and Fabio Zobi [1,*]

[1] Department of Chemistry, Fribourg University, Chemin Du Musée 9, 1700 Fribourg, Switzerland

[2] Haute école d'ingénierie et d'architecture, University of Applied Sciences Western Switzerland HES-SO, Pérolles 80, 1700 Fribourg, Switzerland

*olimpia.mamulasteiner@hefr.ch

*fabio.zobi@unifr.ch

Table of content

^{1}H-NMR spectra of **2-5** – Figures S1-S12 – page S2-S7

IR spectra (solid state) of **2-5** – Figures S13-S14 – page S8

UV-Vis spectra of **2-5** – Figures S15-S16 – page S9

X-Ray π-π interaction of **1** – Figure S17 – Page S9

Homological model Residues in the pocket – Figure S18 – Page S10

Histograms of surrounding amino acids and H-bonds distribution maps for Drug **1-3** and **5** – Figure S19-S25 – Page S11-S14

Table S1. Prescreening AutoDock molecular docking results for different homological MurG models with inhibitors **1**–**5**. – Page S15-S16

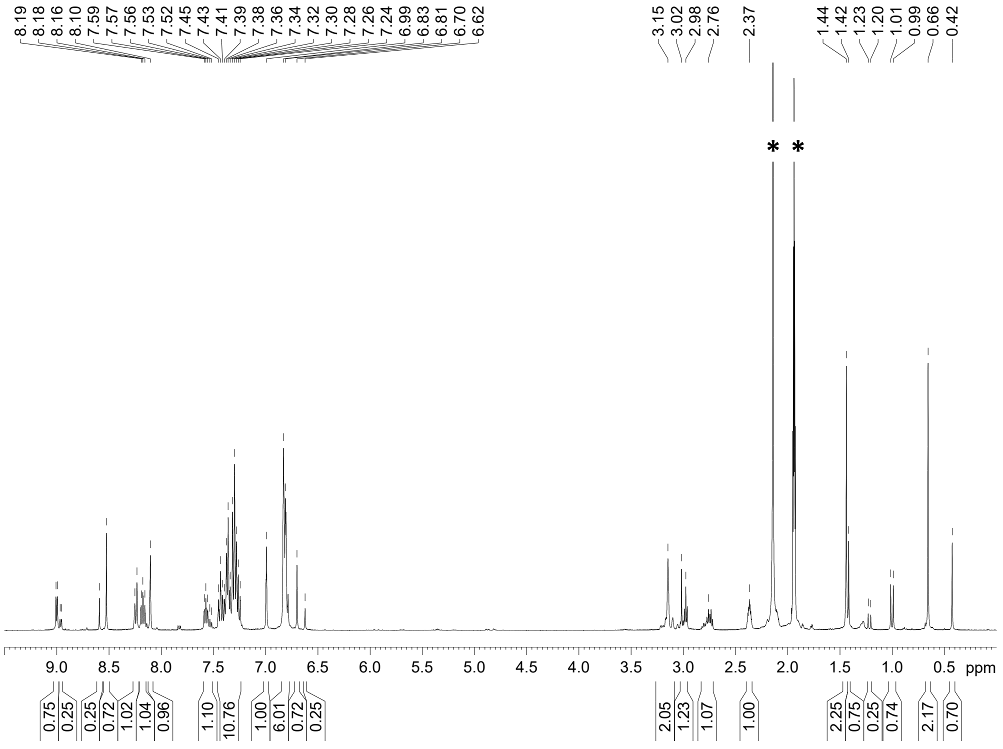

Figure S1. ^{1}H NMR spectrum of a **2a**/**2b** 3:1 mixture in CD_3CN, ✱ = solvent residual peak.

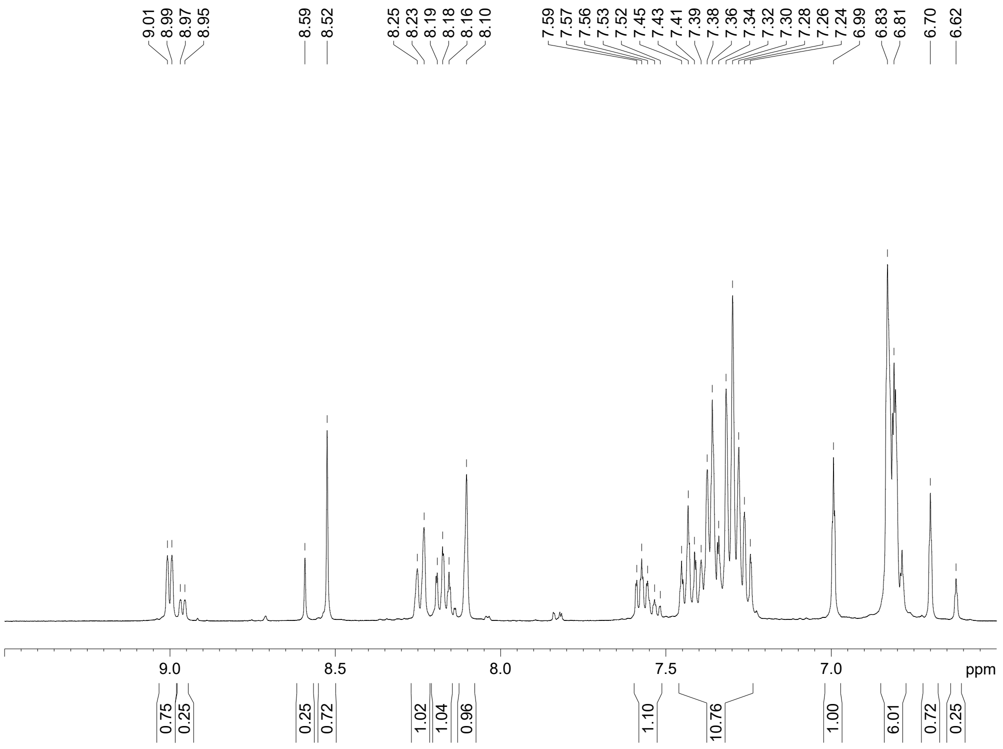

Figure S2. ^{1}H NMR spectrum of a **2a**/**2b** 3:1 mixture in CD_3CN, aromatic region.

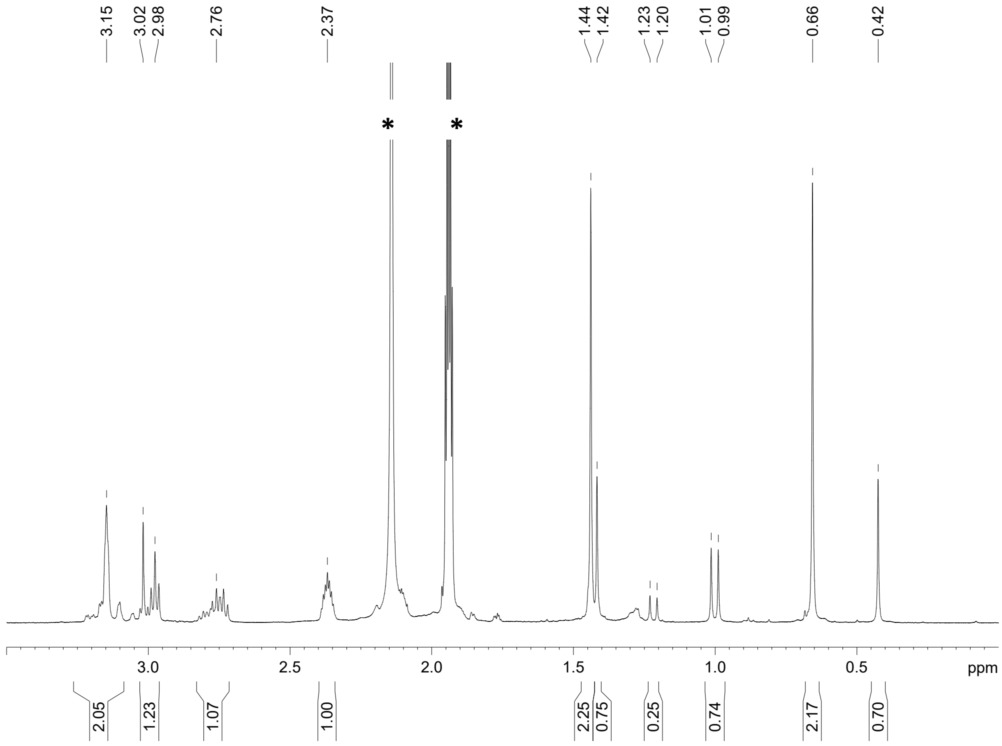

Figure S3. ^{1}H NMR spectrum of a **2a**/**2b** 3:1 mixture in CD_3CN, aliphatic region.

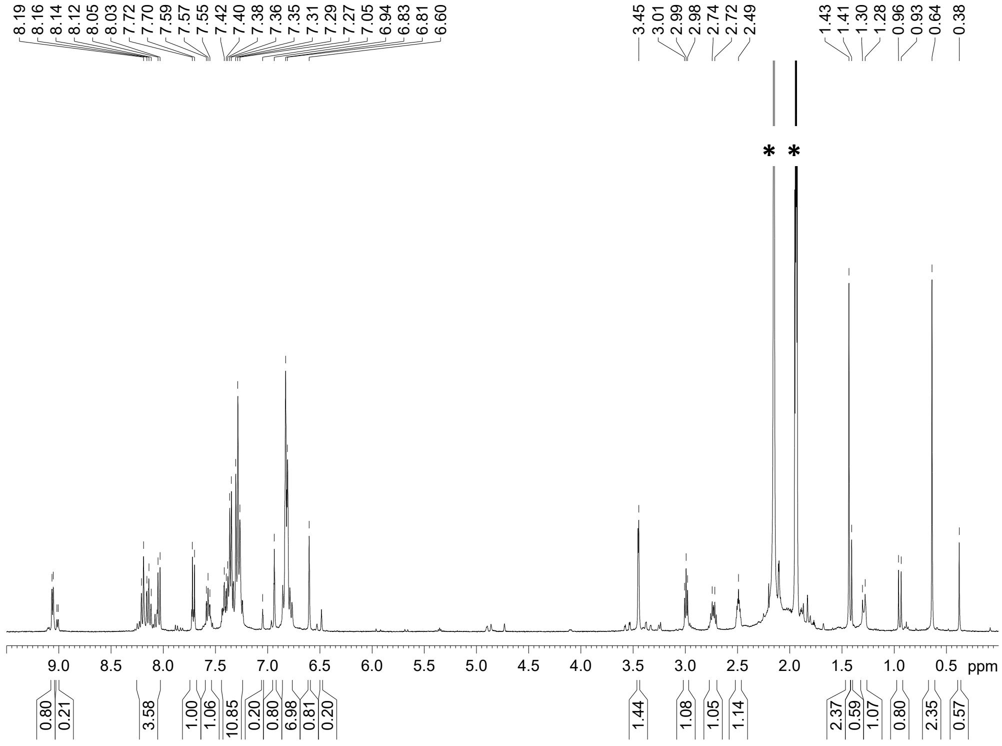

Figure S4. ^{1}H NMR spectrum of a **3a**/**3b** 4:1 mixture in CD_3CN, ✱ = solvent residual peak.

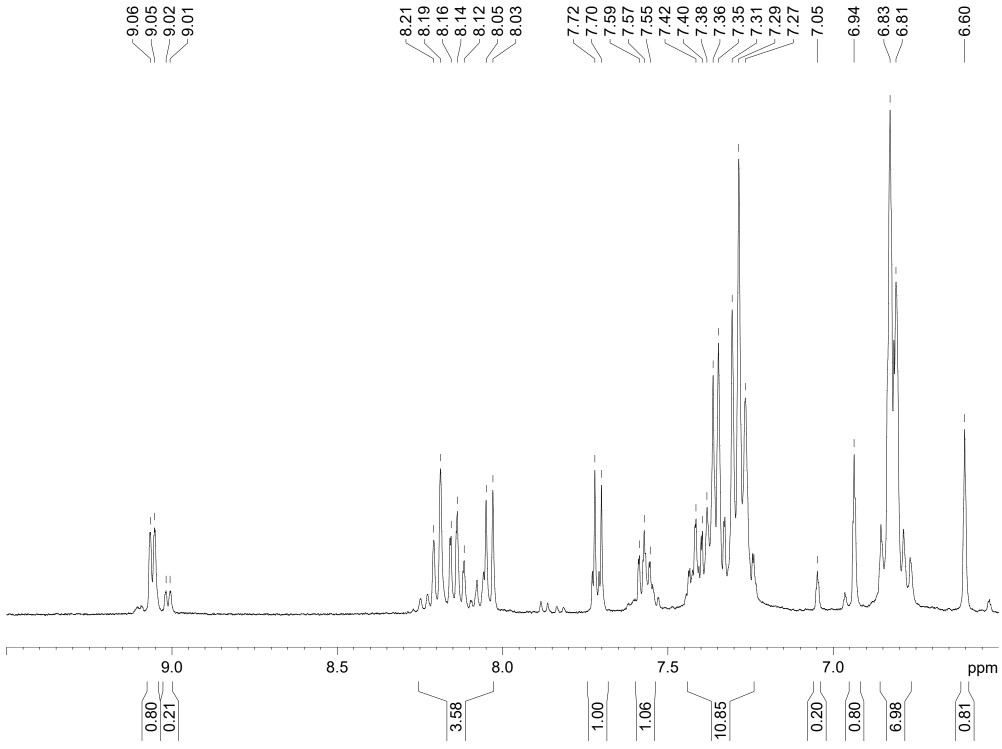

Figure S5. ^{1}H NMR spectrum of a **3a**/**3b** 4:1 mixture in CD_3CN, aromatic region.

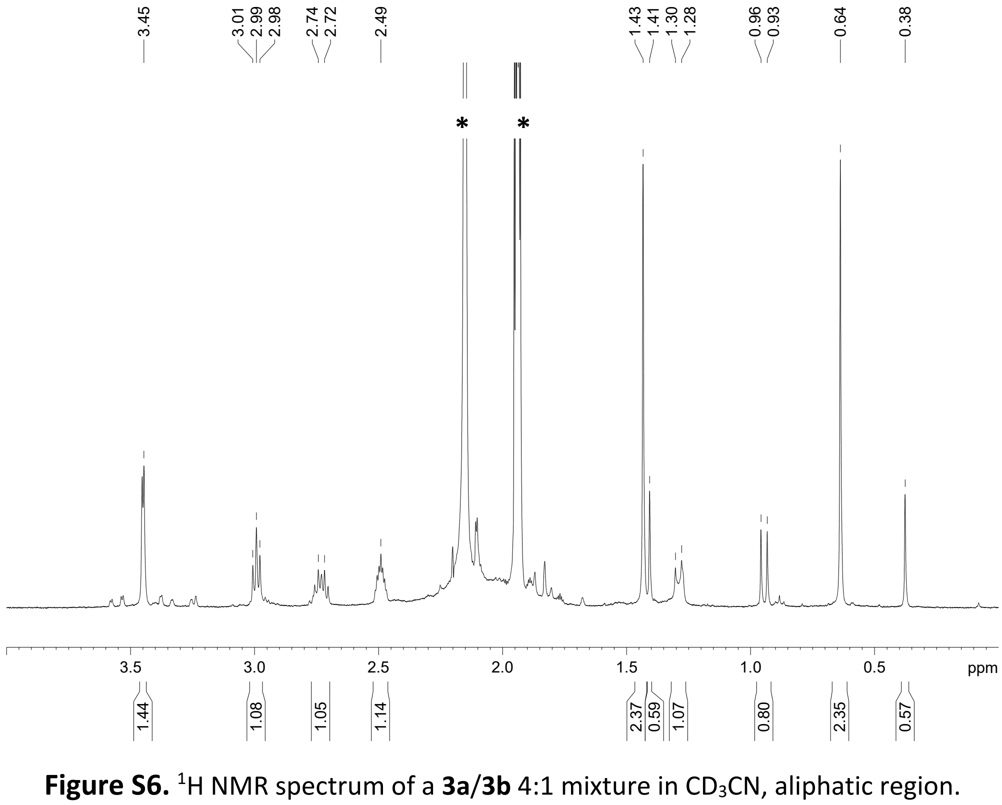

Figure S6. ^{1}H NMR spectrum of a **3a**/**3b** 4:1 mixture in CD_3CN, aliphatic region.

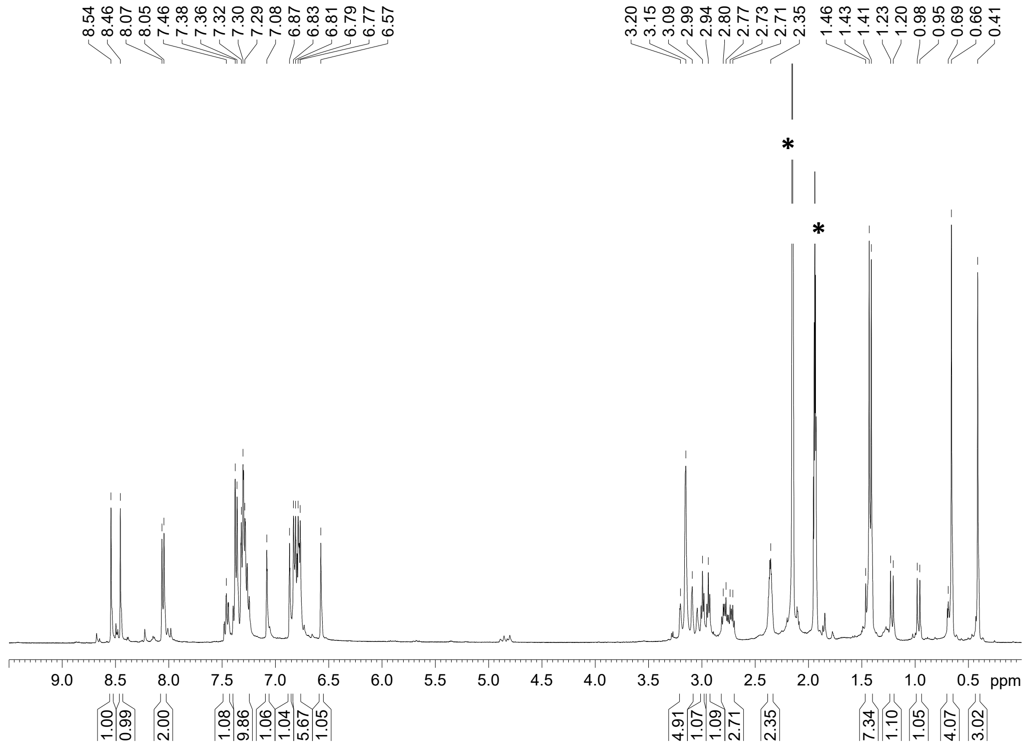

Figure S7. ^{1}H NMR spectrum of **4** in CD_3CN.

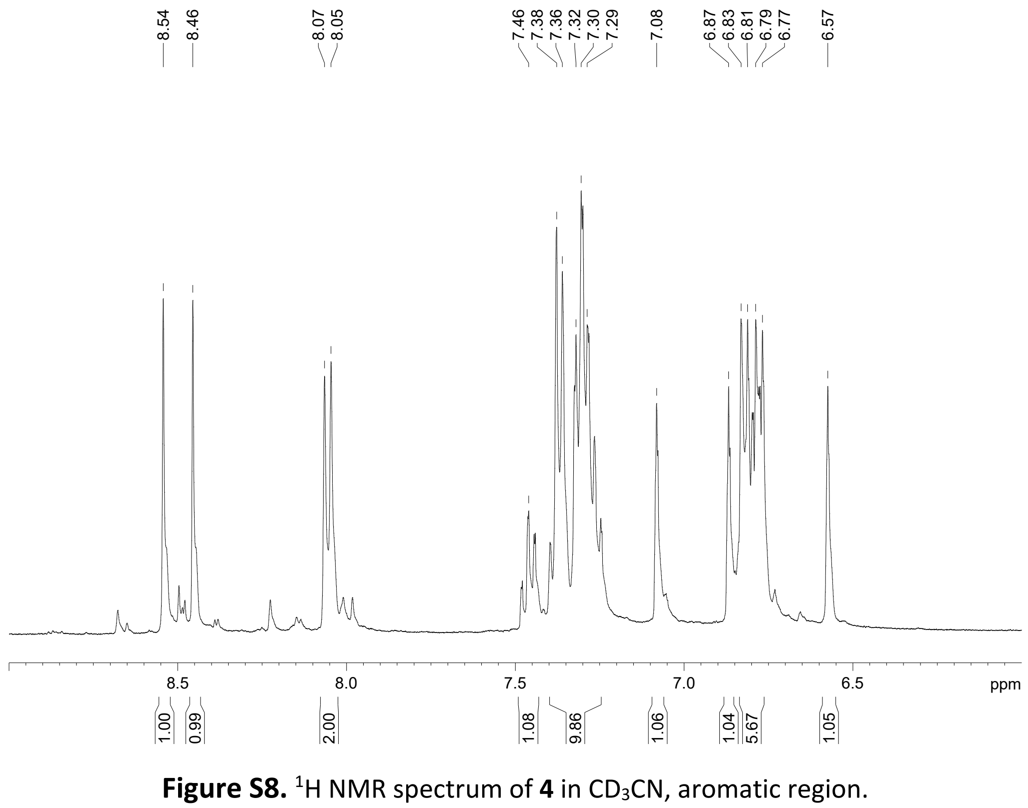

Figure S8. ^{1}H NMR spectrum of **4** in CD_3CN, aromatic region.

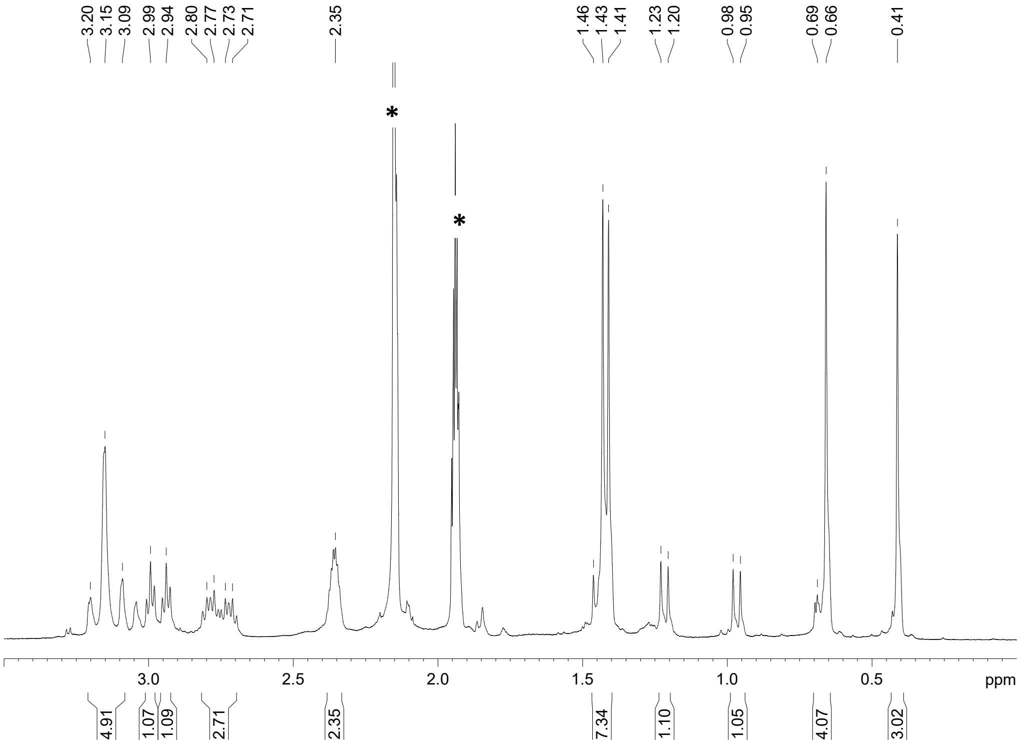

Figure S9. ^1H NMR spectrum of **4** in CD_3CN, aliphatic region.

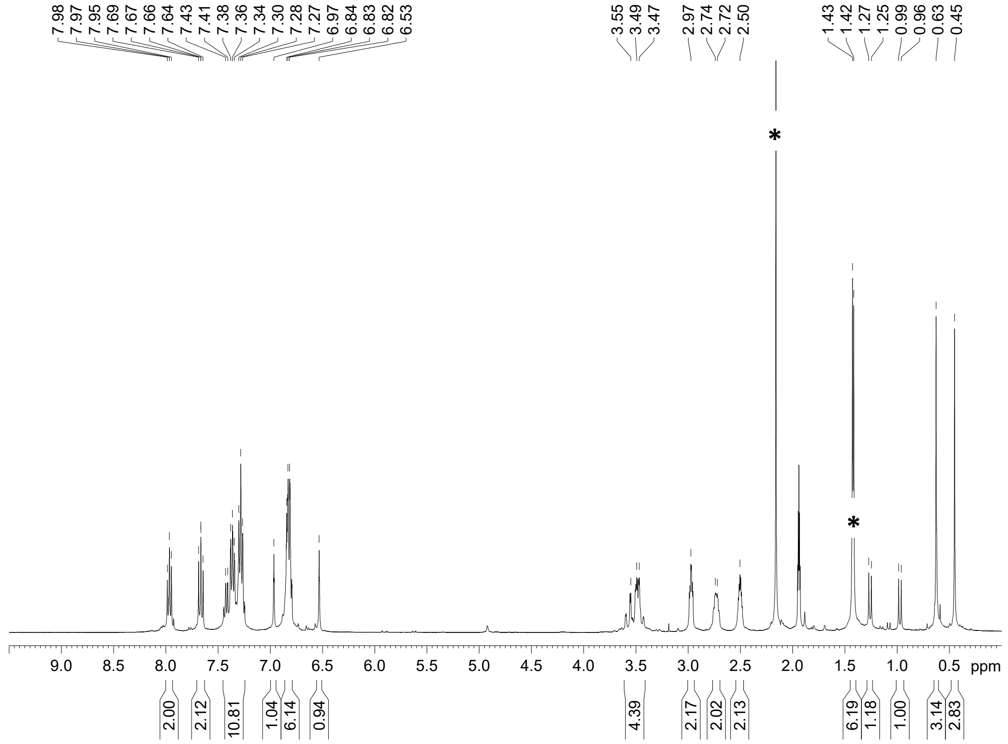

Figure S10. ^1H NMR spectrum of **5** in CD_3CN.

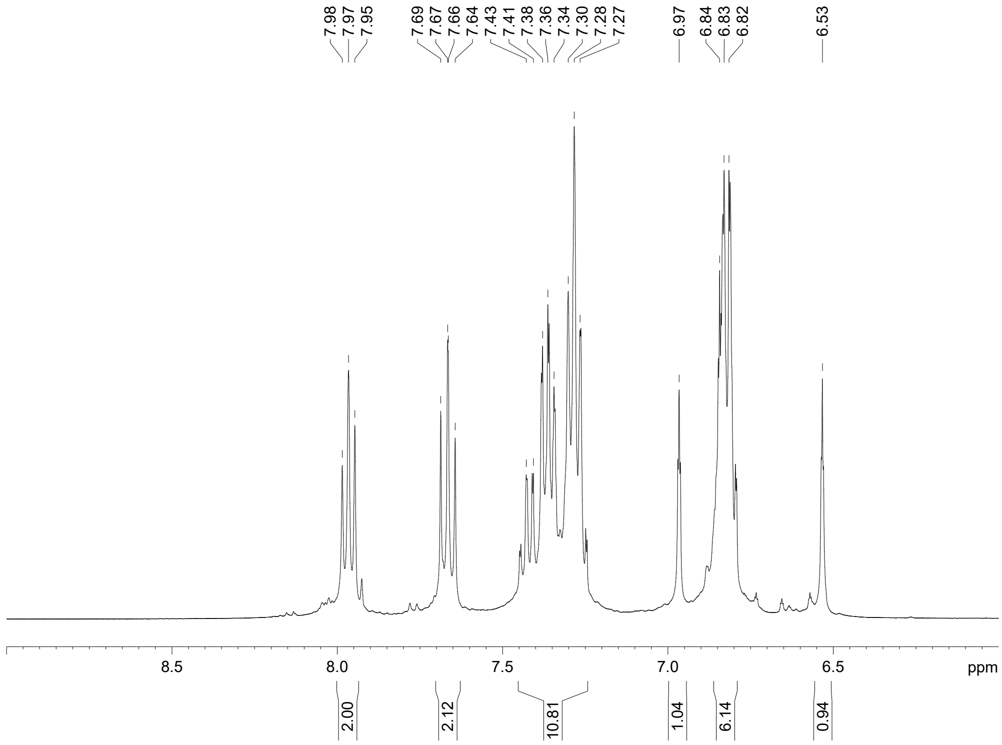

Figure S11. ^{1}H NMR spectrum of **5** in CD_3CN, aromatic region.

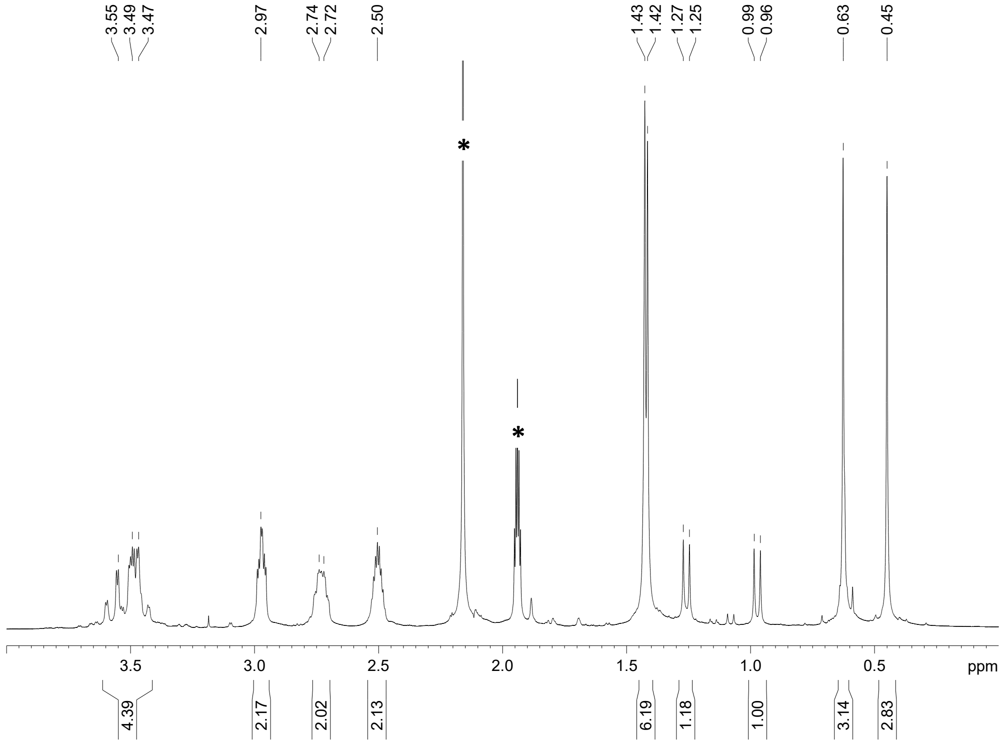

Figure S12. ^{1}H NMR spectrum of **5** in CD_3CN, aliphatic region.

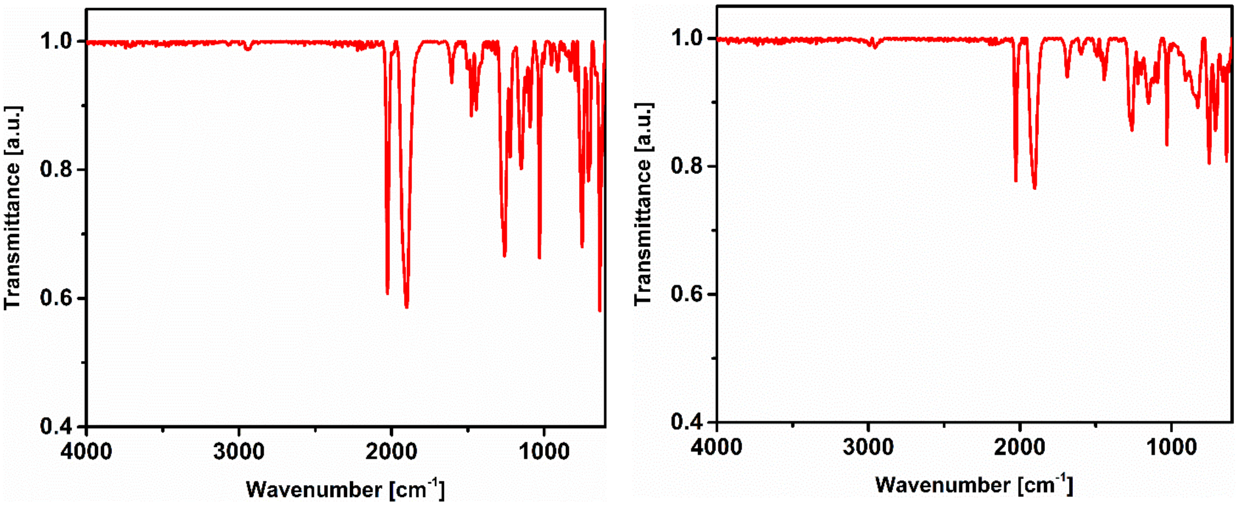

Figure S13. IR spectrum of a **2a/2b** 3:1 mixture (left) and of a **3a/3b** 4:1 mixture.

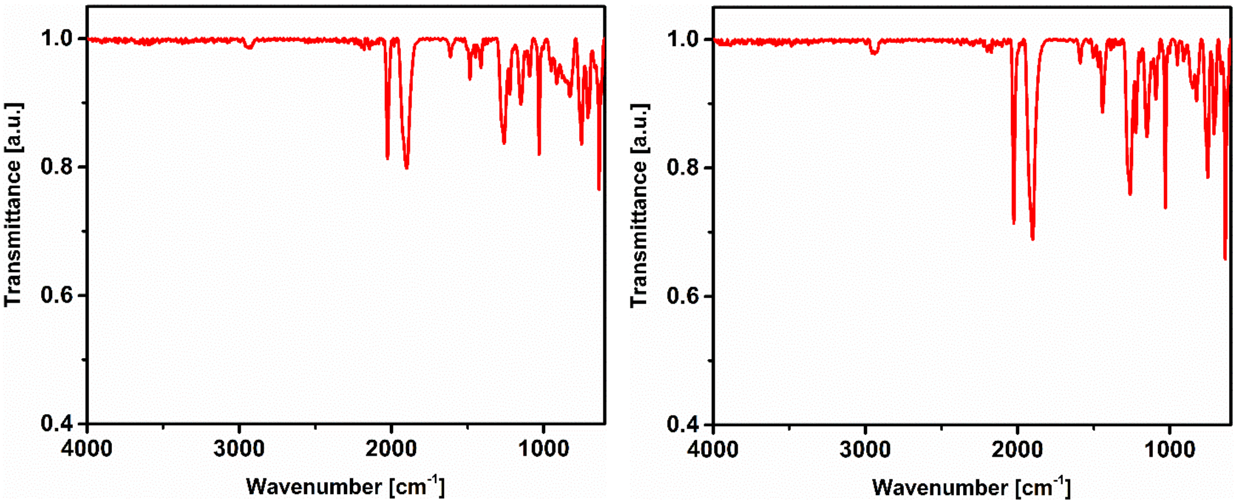

Figure S14. IR spectrum of **4** (left) and **5**.

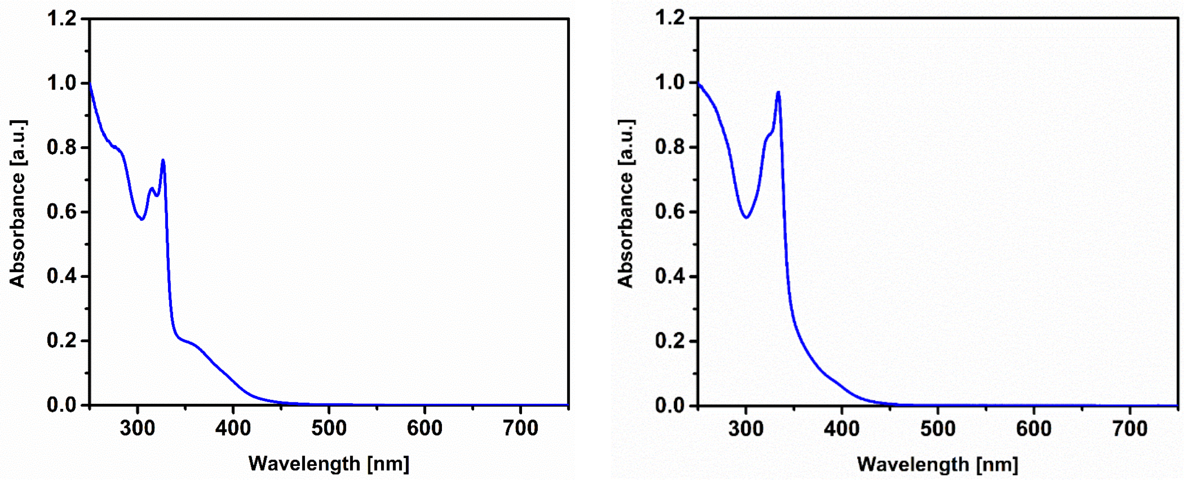

Figure S15. UV-Vis spectrum of a **2a/2b** 3:1 mixture (left) and of a **3a/3b** 4:1 mixture in MeOH.

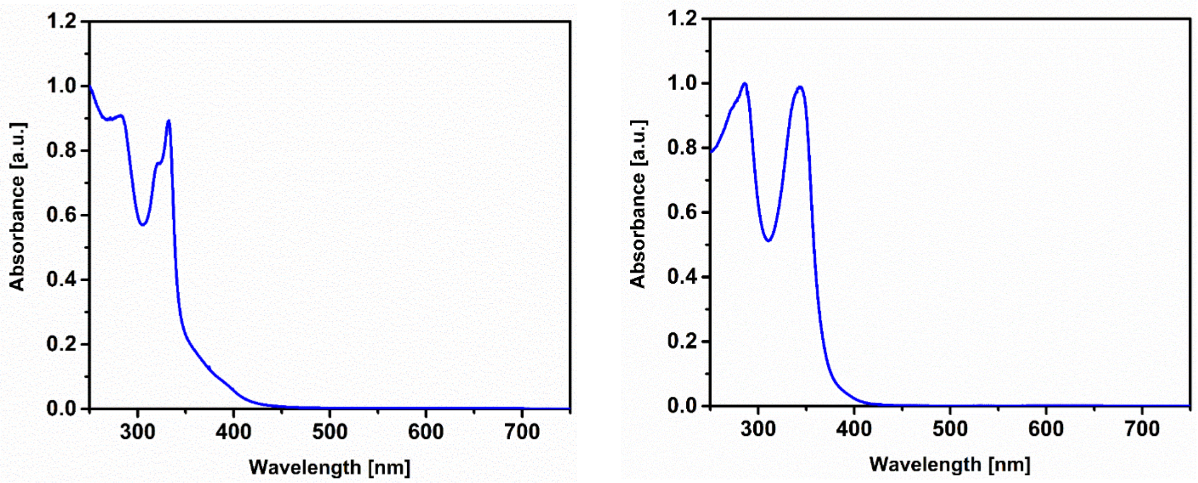

Figure S16. UV-Vis spectrum of **4** (left) and of **5** in MeOH.

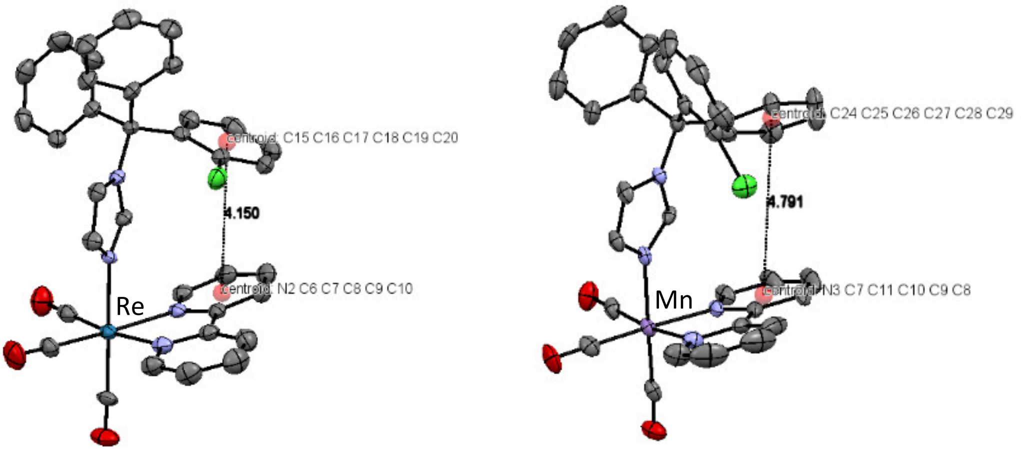

Figure S17. Weak interaction between one of the ctz phenyl rings and one of the aromatic rings of the bpy ligand in the structures of **1** (left) and corresponding Mn complex.

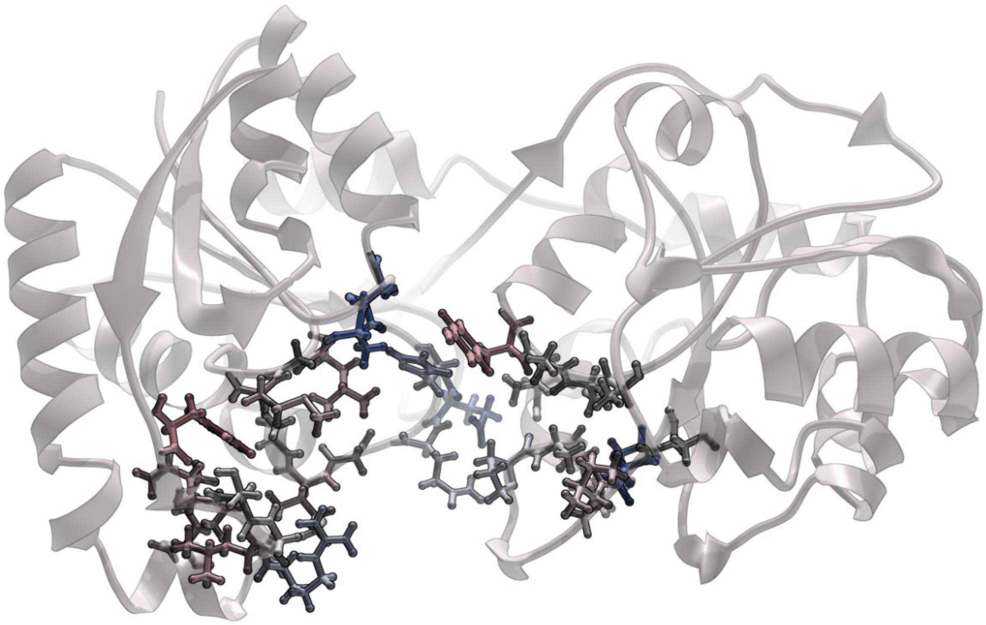

Figure S18. Homological model Residues in the pocket - **Hydrophobic side chain residues** - ALA13 PHE60 ILE63 GLY65 LEU66 ILE71 LEU101 GLY102 MET103 HIS125 GLY129 ILE130 ALA131 ALA249 ALA250 ALA251 ILE270 PRO276 ALA277 VAL280 **Polar but not charged residuals** - THR16 SER64 ARG67 GLU126 GLN127 TYR252 SER268 GLU269

Docking Poses

The depicted docking poses presented in figure 19 to Z, which are conformations of the ligands in the defined binding pocket. The corresponding docking scores were listed in Table 2 in the main text.

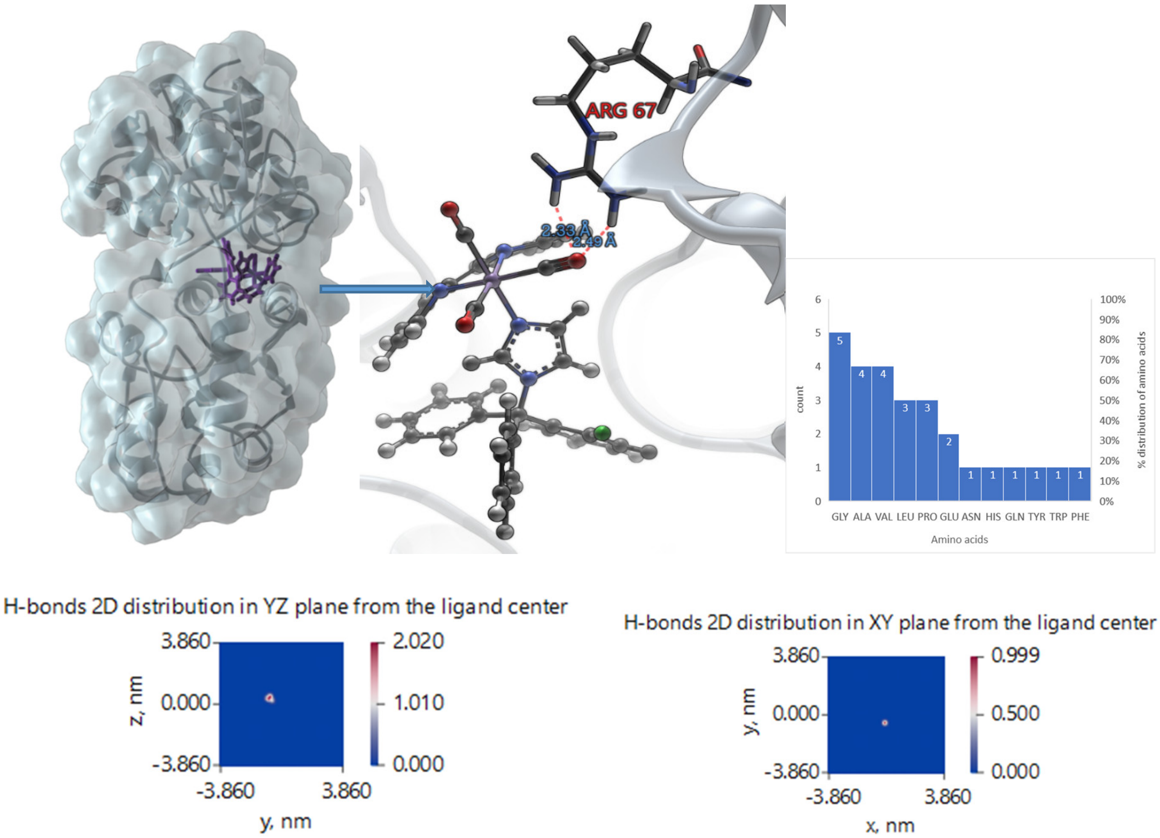

Figure S19. Surrounding amino acids histogram for Drug **1** and 2D distribution bonds in two planes (YZ and XY) for the H-bonds from the center of the ligand.

The geometry of the formed two hydrogen bonds for the carbonyl oxygen in the drug 1 - receptor was depicted at Fig. S19. The displayed 2D map is computed for a certain plane P (xy,xz,yz), which is scaled to the center of the ligand molecule depending of the case.

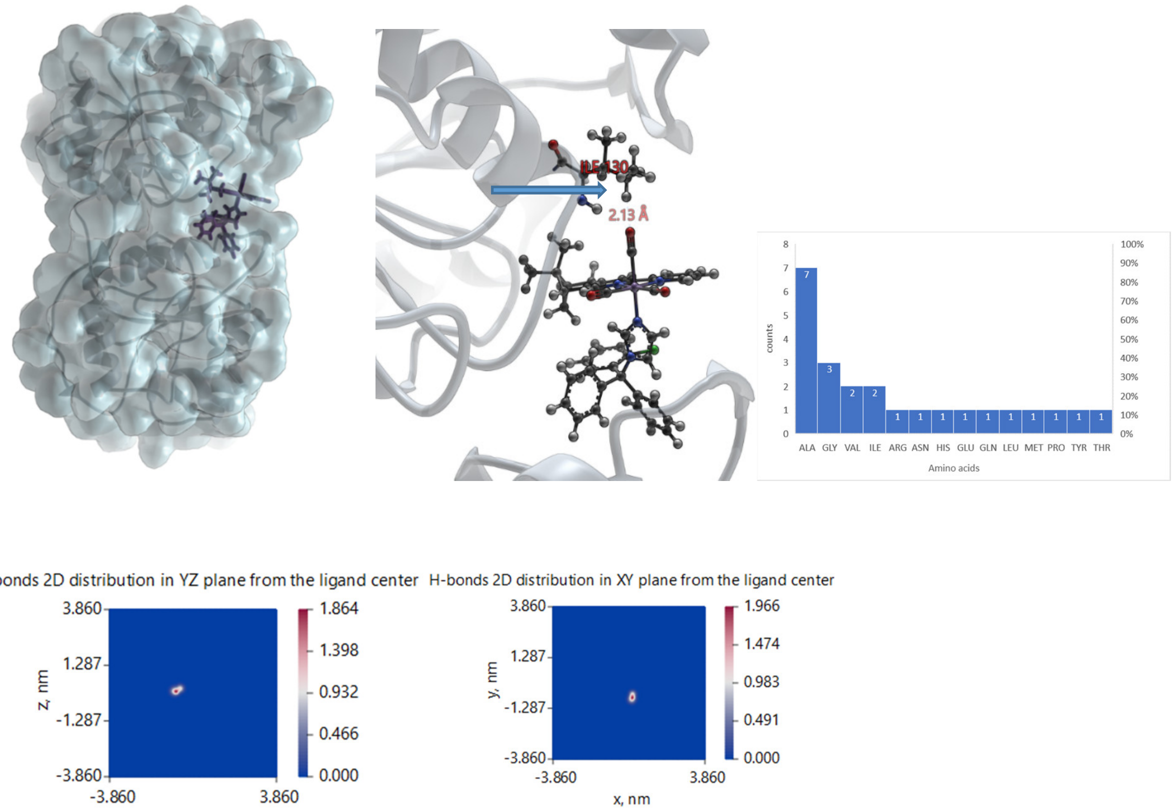

Figure S21. Surrounding amino acids histogram for Drug **2a** and H-bonds distribution maps in two planes (ZY and XY).

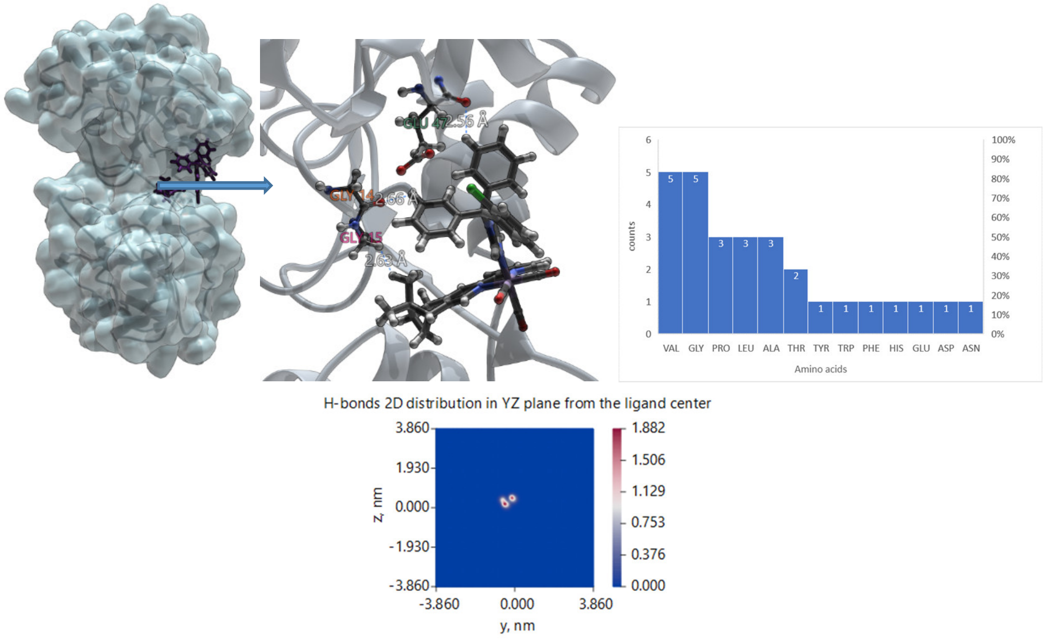

Figure S22. Surrounding amino acids histogram for Drug **2b** and H-bonds distribution map in plane YZ.

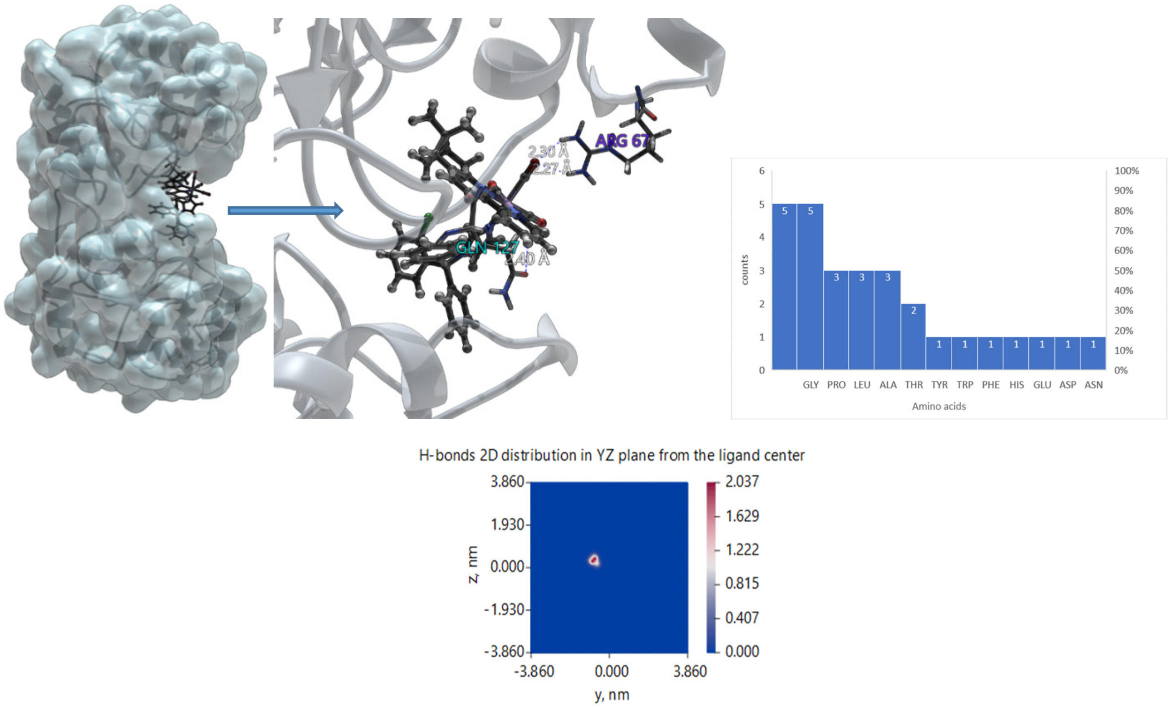

Figure S23. Surrounding amino acids histogram for Drug **3a** and H-bonds distribution map for plane YZ.

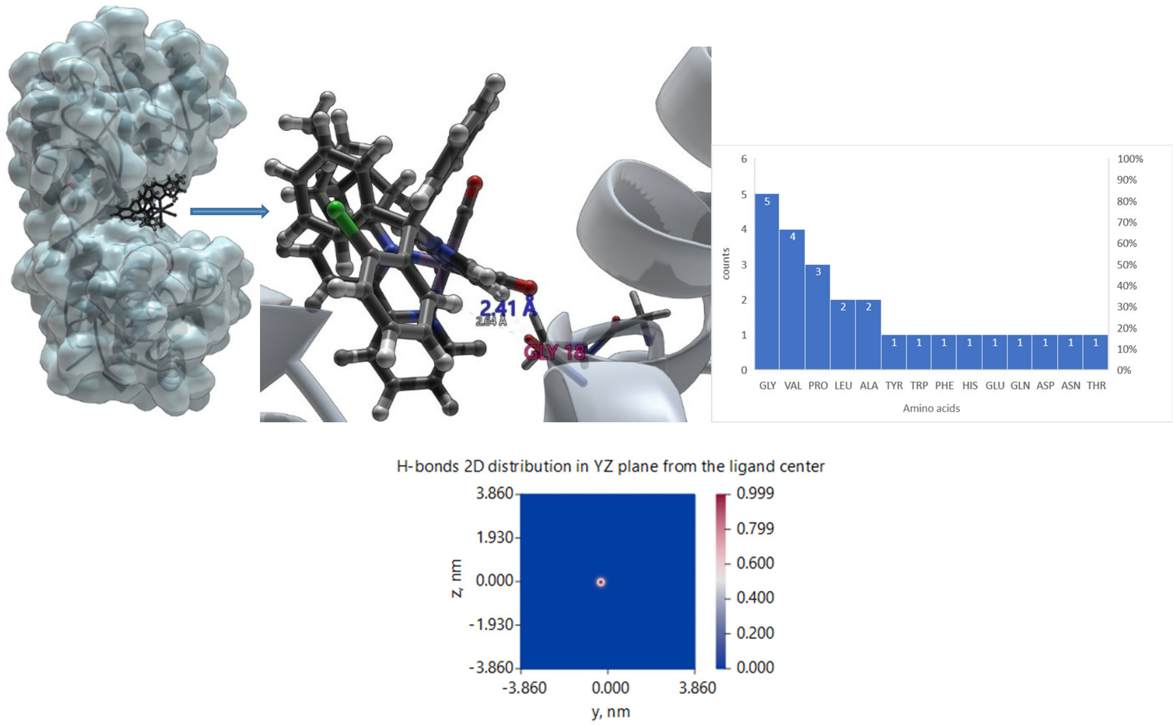

Figure S24. Surrounding amino acids histogram for Drug **3b** and H-bonds distribution map for plane YZ.

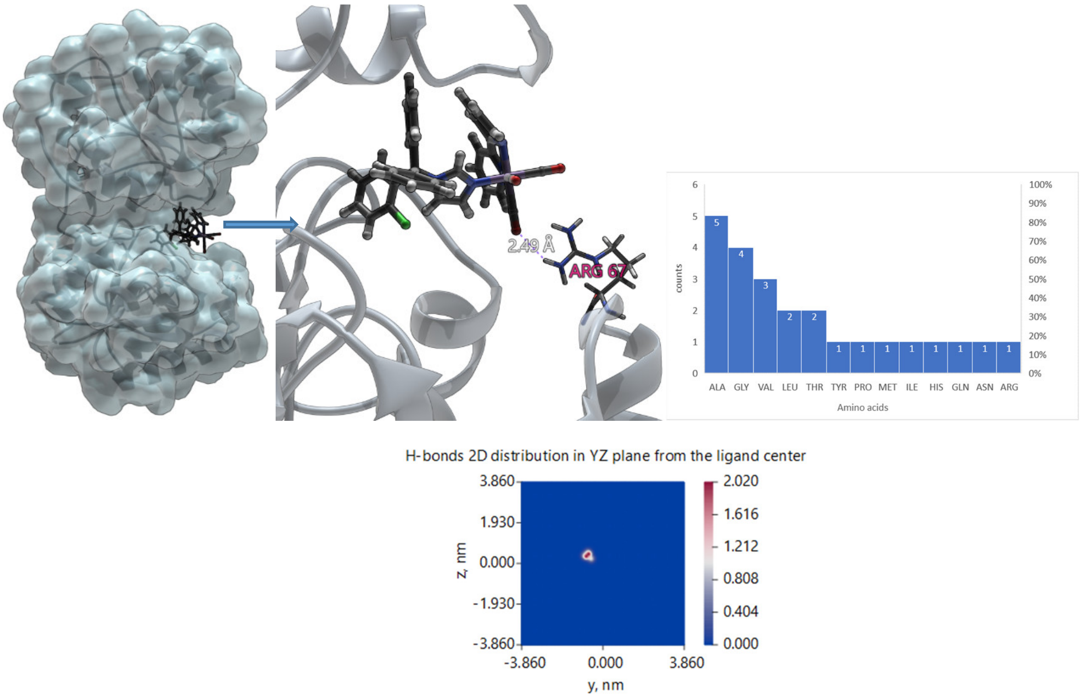

Figure S25. Surrounding amino acids histogram for Drug **3b** and H-bonds distribution map for plane YZ.

Table S1. Prescreening AutoDock molecular docking results for different homological MurG models (fixed receptor) with inhibitors **1–5**.

Receptor	Drug	Affinity Kcal/mol	H-bonds	Receptor's prob. drugability	Detected H-bonds with AA residue
**1NLM**	**1**	-9.2	4	0.982	Asn128, Leu265, Gln288, Asn292
	**2a**	-9.4	0	0.982	
	**2b**	-9.9	3	0.982	Gly17, Gly18, Ser192
	**3a**	-9.3	1	0.982	Ser192
	**3b**	-8.9	0	0.982	
	**4**	-9.9	0	0.982	
	**5**	-9.3	0	0.982	

Receptor	Drug	Affinity Kcal/mol	H-bonds	Receptor's prob. drugability	Detected H-bonds with AA residue
**SaMurG Phyre2**	**1**	-8.9	3	0.993	Gly 101, Asn 124, Ala130
	**2a**	-8.9	1	0.993	Tyr102
	**2b**	-10.3	1	0.993	Gln288
	**3a**	-8.9	1	0.993	Thr129
	**3b**	-9.0	1	0.993	Thr12
	**4**	-8.7	1	0.993	Tyr102
	**5**	-9.7	2	0.993	Thr12, Tyr102

Receptor	Drug	Affinity Kcal/mol	H-bonds	Receptor's prob. drugability	Detected H-bonds with AA residue
**SaMurG UniProt**	**1**	-8.2	1	0.986	Glu167
	**2a**	-9.0	1	0.986	Thr11
	**2b**	-8.7	0	0.986	
	**3a**	-9.4	0	0.986	
	**3b**	-9.4	2	0.986	Thr11, Arg287
	**4**	-9.7	0	0.986	
	**5**	-8.9	0	0.986	

Receptor	Drug	Affinity Kcal/mol	H-bonds	Receptor's prob. drugability	Detected H-bonds with AA residue
**SaMurG MOE**	**1**	-8.5	0	0.712	
	**2a**	-9.1	0	0.712	
	**2b**	-9.3	1	0.712	Asn161
	**3a**	-8.9	1	0.712	Arg67
	**3b**	-9.1	0	0.712	
	**4**	-10.1	0	0.712	
	**5**	-9.7	0	0.712	

Receptor	Drug	Affinity Kcal/mol	H-bonds	Receptor's prob. drugability	Detected H-bonds with AA residue
SaMurG freefold	**1**	-9.0	2	0.986	Asn124, Gln288
	**2a**	-9.2	1	0.986	Thr129
	**2b**	-9.8	1	0.986	Asn124
	**3a**	-9.4	1	0.986	Gln288
	**3b**	-10.9	1	0.986	Asn124
	**4**	-9.9	2	0.986	Thr12, Gln288
	**5**	-11.7	2	0.986	Asn124, Gln288
